# Commercial head-mounted display virtual reality for upper extremity rehabilitation in chronic stroke: a single-case design study

**DOI:** 10.1186/s12984-020-00788-x

**Published:** 2020-11-23

**Authors:** Mattias Erhardsson, Margit Alt Murphy, Katharina S. Sunnerhagen

**Affiliations:** 1 Institute of Neuroscience and Physiology, Clinical Neuroscience, Rehabilitation Medicine, Sahlgrenska Academy, University of Gothenburg, Per Dubbsgatan 14, 3rd Floor, 41345 Gothenburg, Sweden; 2 Institute of Biomedicine, Medical Biochemistry and Cell Biology, Sahlgrenska Academy, University of Gothenburg, Medicinaregatan 9 A, 413 90 Gothenburg, Sweden

**Keywords:** Stroke, Upper extremity, Movement, Activity, Rehabilitation, Virtual reality, Head-mounted display, Kinematics, Video games

## Abstract

**Background:**

Rehabilitation is crucial for maximizing recovery after stroke. Rehabilitation activities that are fun and rewarding by themselves can be more effective than those who are not. Gamification with virtual reality (VR) exploits this principle. This single-case design study probes the potential for using commercial off-the-shelf, room-scale head-mounted virtual reality for upper extremity rehabilitation in individuals with chronic stroke, the insights of which can inform further research.

**Methods:**

A heterogeneous volunteer sample of seven participants living with stroke were recruited through advertisement. A single-case design was employed with a 5-week baseline (A), followed by a 10-week intervention (B) and a 6-month follow-up. Upper extremity motor function was assessed with validated kinematic analysis of drinking task. Activity capacity was assessed with Action Research Arm Test, Box and Block Test and ABILHAND questionnaire. Assessments were done weekly and at follow-up. Playing games on a VR-system with head-mounted display (HTC Vive) was used as rehabilitation intervention. Approximately 300 games were screened and 6 tested. Visual analysis and Tau-U statistics were used to interpret the results.

**Results:**

Visual analysis of trend, level shift and overlap as well as Tau-U statistics indicated improvement of Action Research Arm Test in six participants. Four of these had at least a moderate Tau-U score (0.50–0.92), in at least half of the assessed outcomes. These four participants trained a total of 361 to 935 min. Two out of four participants who were able to perform the drinking task, had the highest training dose (> 900 min) and showed also improvements in kinematics. The predominant game played was Beat Saber. No serious adverse effects related to the study were observed, one participant interrupted the intervention phase due to a fall at home.

**Conclusions:**

This first study of combining commercial games, a commercial head-mounted VR, and commercial haptic hand controls, showed promising results for upper extremity rehabilitation in individuals with chronic stroke. By being affordable yet having high production values, as well as being an easily accessible off-the-shelf product, this variant of VR technology might facilitate widespread adaption. Insights garnered in this study can facilitate the execution of future studies.

*Trial registration* The study was registered at researchweb.org (project number 262331, registered 2019-01-30, https://www.researchweb.org/is/vgr/project/262331) prior to participant enrolment.

## Background

Post-stroke sequelae can encompass any number of domains associated with cerebral function, including motor, sensory, language and cognitive functions. Upper extremity motor function is affected in approximately 50% of patients early after stroke [1]. About 1/3 of those with early upper extremity impairment will achieve full dexterity in the chronic stage of recovery [2] Rehabilitation is crucial for maximizing recovery from neurological conditions, including stroke. Most of the rehabilitation interventions are concentrated to the first 3 to 6 months after stroke, although the need remains for years to come [3]. Rehabilitation activities that are more engaging, e.g. virtual reality (VR), can be more effective compared to conventional rehabilitation [4, 5]. VR has been shown to improve upper extremity functioning when used in addition to conventional rehabilitation [6, 7]. A rehabilitation activity that is enjoyable can also enhance adherence and long-term use. Gaming augmented with visual and audio feedback exploits neurophysiological reward mechanisms e.g. by engaging dopaminergic reward systems, which can enhance brain plasticity [8, 9].

The VR research field is heterogeneous and has been likened to the “Wild West” [10]. VR systems within the rehabilitation context can be grouped into systems that are customized for rehabilitation [11], and those who are off-the-shelf systems for a broader entertainment market [12]. The advantage of customized systems developed for rehabilitation purposes is that they follow rehabilitation principles and can thus be intrinsically useful for rehabilitation. Commercial off-the-shelf systems on the other hand can be both more economical, entertaining as well as have a higher product quality, but in turn do require adaptation to find its place as a rehabilitation tool.

Although the layman might term only head-mounted displays (HMD) as VR, console games [13–16], 3D-monitors [17], and HMD [11, 18–20] are all denoted as VR within academic literature [10, 21]. On this spectrum, HMD represents the most immersive VR technology. Literature pertaining to VR, based on HMD for upper extremity stroke rehabilitation, is limited [21]. Rehabilitation approaches tested with HMD VR include both custom hardware and software [11], as well as off-the-shelf hardware with custom software [19, 20]. However, combining both off-the-shelf hardware and software seems to be unexplored ground in the field of stroke rehabilitation.

Upper extremity rehabilitation can benefit from technology that stimulate involved neurological pathways [22]. These pathways can be stimulated in room-scale HMD VR systems with haptic hand controls, where sensors track hand and head movement in 6 degrees of freedom. The market for commercial off-the-shelf room-scale VR was dominated by Oculus Rift, HTC Vive and PlayStation VR at the start of this study (early spring 2019). Among these, HTC Vive was brought to bear for this study as it was simpler to set up and use than Oculus Rift and had a far greater repertoire of games available than PlayStation VR. A monitor displays approximately what the user sees in the HMD, facilitating demonstrations by, and support from accompanying personnel.

The overall aim of this study was to explore what potential commercial off-the-shelf, head-mounted display, room-scale virtual reality has for chronic stroke rehabilitation with focus on upper extremity functioning. The results can help lay the foundations for future larger-scale studies. The study aimed also to provide further insights on which HMD-VR games can be suitable for people with chronic stroke, who might benefit most, and which outcome measures might be most suitable for evaluation.

## Methods

The SCRIBE reporting guideline checklist was utilized for this article [23]. The study was registered at researchweb.org (project number 262331) prior to participant enrolment [24].

## Study design

Initial forays into novel interventions require development and piloting before larger randomization trials [25, 26]. Thus, a multiple-participant, single-case design was chosen since it is sensitive to individual improvement and is of appropriate scope for a small-scale rehabilitation study [27, 28]. The single-case design employed in this study consisted of a baseline phase (phase A), an intervention phase (phase B), as well as a 6-month follow up. Baseline included 5 assessments performed once a week. During the 10 weeks intervention phase the assessments were performed once a week. While this non-randomized, non-blinded study design is unable to determine causal relationships, it can demonstrate temporal correlations.

### Participants

**Fig. 1:**
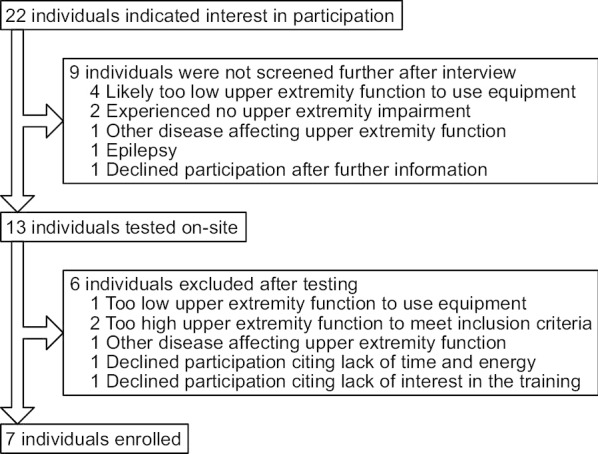
Participant recruitment flowchart

Recruitment was conducted through advertisement at patient organizations and support groups, local health-care providers, as well as informing participants from other studies. This resulted in a volunteer sample of 7 individuals with chronic stroke (Fig. 1). Individuals who indicated interest were interviewed to determine if it was plausible they would fulfil inclusion/exclusion criteria. If the person didn’t think it was possible to somehow hold an object like a remote control and press any button with the affected hand, then this was interpreted as likely having too low upper extremity function. If not excluded by the interview, a physical visit was booked to confirm the inclusion according to defined criteria.

The inclusion criteria were: stroke at least 6 months prior and a residual upper extremity deficit identified by at least one of the following criteria: Fugl-Meyer Assessment of Upper Extremity motor score ≤ 60 [29], Action Research Arm Test (ARAT) score ≤ 51 [30], Box and Blocks Test (BBT) at least 6 blocks less compared to normative value and the non-affected hand [31, 32]. Diagnosed with, or under investigation for, any other condition than stroke that affects the upper extremity function, not being able to adhere to protocol or use the equipment, e.g. due to having too low upper extremity motor function or being unable to visit the site at least once a week for 15 weeks entailed study exclusion. Finally, to err on the side of caution of the HTC Vive safety instructions, having a history of photosensitive seizures or a pacemaker also excluded participation.

### Outcome measures

A battery of recommended clinical and kinematic assessments with strong psychometric properties covering body function and activity domains according to International Classification of Functioning and Health (ICF) were used to assess upper extremity functioning [33–35]. Clinical background data were collected through interviews and medical records. All assessments were administered by a trained medical researcher in a random order within the assessment session. During the intervention phase, the assessments took place prior to the training session when they occurred on the same day. Weekly time spent on assessments varied within the approximate range of 15–60 min between participants. Assessments took place in a research facility close to the University hospital.

### Activity domain outcome measures

Action Research Arm Test (ARAT) evaluates the upper extremity activity capacity and includes 19 items divided into four subtests (grasp, grip, pinch, gross movement) [36, 37]. A maximum score of 57 in ARAT indicates full capacity, and the minimally clinically important difference (MCID) is 5.7 [30]. The Box and Blocks Test (BBT) assesses gross motor ability to grasp and move the largest number of small wooden cubes (2.5 cm) from one box to another in 1 min [32], and the minimally detectable change (MDC) is 5.5 [31]. The ABILHAND questionnaire evaluates the person’s perceived difficulty of performing bimanual daily activities [38]. The score is expressed as logits after a Rasch based conversion and the values range from approximately -6 to 6. A score of 0 means that most of the activities are difficult to perform, and the MCID is 0.26–0.35 [39].

### Body function domain outcome measures—kinematics

Validated and recommended kinematic measures of movement time, smoothness and compensatory trunk displacement, calculated from a 3D movement analysis of the drinking task, were used to evaluate changes in movement performance and quality [35, 40]. These 3 measures are valid, reliable and sensitive to change in stroke population and cover the key elements of motor deficits in stroke [41–44]. Kinematic data was acquired with a 5-camera high speed (240 Hz) motion capture system (Qualisys AB, Gothenburg, Sweden). The cameras emit infra-red light that is reflected by the circular markers placed on the anatomical landmarks on the body. The 8 markers were placed on the tested hand (III metacarpophalangeal joint), wrist (styloid process of ulna), elbow (lateral epicondyle), on both shoulders (acromion), trunk (sternum), forehead and the drinking cup [40]. Kinematic data was analyzed and filtered (6-Hz Butterworth filter) in the Matlab software (R2019B, The Mathworks Inc). The drinking task included reaching and grasping the glass, lifting it to the mouth and drinking a sip of water, placing the glass back on the table and returning the hand back on the edge of the table. The starting position was standardized to the body size and the glass was positioned at 30 cm distance from the table edge, which was within the reach of the hand when the back was against the backrest of the chair. The trunk was not restrained, and the participants were instructed to perform the task with the affected arm in a self-paced speed as naturally as possible. After few familiarization trials the task was repeated 10 times with approximately 5 s rest between each trial. The mean of 10 trials was used as test result.

Movement time was defined as time required to complete the entire drinking task. The start and end of the movement was identified from the point where the hand marker surpassed the 2% of the maximum velocity of reaching or returning phase, respectively [40, 41]. The reference value for healthy controls for movement time of the drinking task is 6.28 s (SD 0.98) as reported previously [35]. Movement smoothness was defined as the number of movement units (NMU) identified from the tangential velocity profile of the hand marker. A movement unit was defined as the difference between a local minimum and the next maximum velocity value that exceeded the amplitude limit of 20 mm/s, where the time between two subsequent peaks had to be at least 150 ms. The minimum possible value for NMU was 4 including one predominant peak for each movement phase (reach, transport forward, transport back and return of the hand). The reference value for healthy controls for movement smoothness is 6.0 units (SD 1.0) [35]. Trunk displacement was defined as maximal forward displacement of the sternal marker in the sagittal plane from the initial position during the entire task. The reference value for healthy controls for trunk displacement is 3.3 cm (SD 1.6) [35].

### Additional clinical assessments

In order to keep the assessment time short compared to training time the additional assessments of sensorimotor function were only performed before and after intervention as well as at 6 months follow-up. The Fugl-Meyer Assessment-Upper Extremity (FMA-UE) including assessment of sensation, range of motion and pain was used [45]. Upper extremity spasticity at the elbow and wrist joint was assessed by the modified Ashworth Scale [46]. Physical activity level was assessed by Saltin-Grimby Physical Activity Level Scale (SGPALS) at baseline and at 6-moths follow-up [47].

### Intervention

The intervention consisted of playing VR games on the HTC Vive (HTC Corporation, New Taipei City, Republic of China). The intervention took place in a dedicated VR-room at the research facility. The participants themselves booked access to the system. They were encouraged to play as much as possible. A researcher was present at every training session. During each session the researcher noted what application(s) the participant used and how long these were used. Other observations, participants’ thoughts and experiences and possible adverse effects were recorded as field notes. Participants scored their perceived physical exertion after every training session on Borg Rating of Perceived Exertion, of which a median value for all sessions was calculated (Table 1) [48].

**Table 1:** Characteristics of participants

Participant ID	P1	P2	P3	P4	P5	P6	P7
Age	65	64	48	53	69	74	51
Sex	M	M	M	F	M	F	M
Type of stroke	Inf	Hem	Inf	Inf	Inf	Inf	Inf
Location of stroke	SCA lacunar	Basal ganglia internal capsule	MCA	Putamen corona radiata	MCA Pons	Basal ganglia	Multiple cerebral
Time since stroke, years	1	3	0.6	1	6 and10	4	2
Reperfusion treatment	No	No	Yes	Yes	No	No	No
Dominant arm	Right	Left	Right	Right	Right	Right	Right
Affected arm	Left	Left	Right	Left	Right	Left	Left
Physical activity level (1 to 4)	2	2	2	2	2	2	2
Number of baseline assessments	5	4	5	5	5	5	5
Number of assessments during intervention	10	9	10	7	10	7	3
Number of training sessions	27	9	22	9	21	9	4
Total training time, min	739	375	935	361	915	198	105
Borg RPE (6 to 20), median	15	16	17	17	18	15	12

*P* participant, *F* female, *Inf* infarction, *Hem* hemorrhage, *SCA* superior cerebellar artery, *MCA* middle cerebral artery, *SGPALS* Physical activity level was assessed by Saltin-Grimby Physical Activity Level Scale, *RPE* rating of perceived exertion

Approximately 300 games out of the 3000 available for HTC Vive were screened for its potential to be used in the study. Screening was done by the first author (ME), who has extensive gaming experience, by systematically browsing the Steam store page. VR games were sorted by falling popularity and about 300 of the most popular games were assessed for their suitability. If a game was not disregarded outright the first author looked at gameplay clips, read reviews, and if available played demo versions. Six games were promising enough to be downloaded and tested. Five were made available to the participants, as one game was deemed to present an unacceptable risk of falling. Participants decided themselves, but with guidance, what and how to play. Beat Saber was the overwhelmingly most common game to see play, NVIDIA VR funhouse saw some play, while the other games saw little to no play. Beat Saber is a rhythm-based game while NVIDA VR funhouse consists of several carnival-style mini-games. See Additional file 1 for further details about the games and the screening process.

**Fig. 2:**
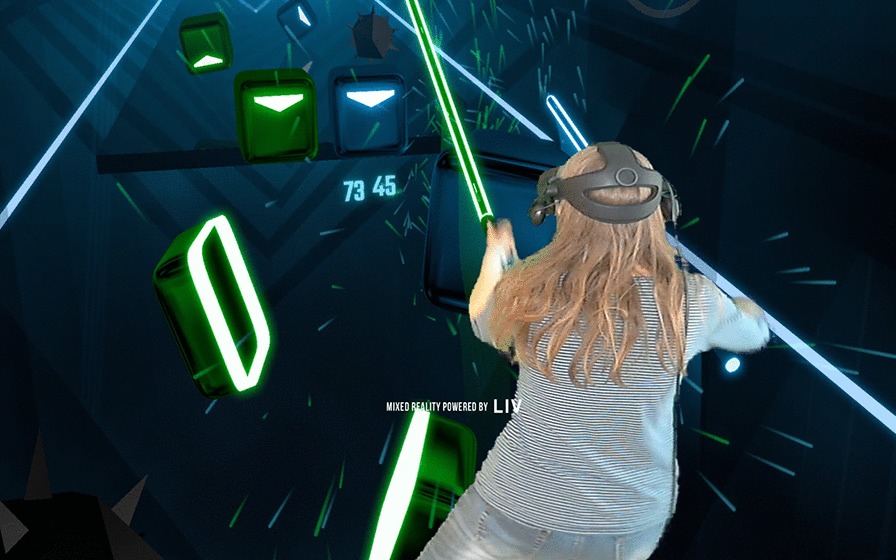
Screenshot of Mixed Reality footage of a participant playing Beat Saber. Mixed Reality was provided by the 3rd party software LIV, and was captured with OBS Studio

In Beat Saber, the user has one lightsaber in each hand, which is used to cut blocks to the sound of music. The gameplay forces the player to use both hands. The difficulty can be adjusted in a massive range, which stretches from beyond the best of human capacity to a negligible difficulty level. There is a risk of sensory overload even at the lowest difficulties, although lowering the volume and enabling options reducing special effects can somewhat mitigate this. The gameplay itself do not require the user to press buttons, allowing individuals with severe upper extremity impairment to play it e.g. through attachment of the hand controller to the hand with Velcro straps. It was designed to be played standing but can be played sitting. See Fig. 2 for a visualization of a participant playing Beat Saber.

### Statistical and visual analysis

Visual analysis is the accepted norm within single-case design studies, but other than that there’s no consensus as to what other of the multitude of available data analysis methods that should be used [27, 28, 49–52]. Visual analysis was conducted as follows: (i) determine stable baseline trend; (ii) to compare trends, levels and variability within and between phases; and (iii) to assess overlap and consistency of patterns [49]. Immediacy of effect [49] was not assessed since rehabilitation interventions are not expected to yield immediate effects after initiation of the intervention. The visual analysis was first conducted independently by two authors (ME, MAM) and then jointly to reach a consensus.

Of available analytical statistics techniques for single-case design data [53], the post-hoc statistical analysis of Tau-U was deemed the best fit for this study’s data [54]. Tau-U can adjust for the baseline trends evident in our data, and unlike the majority of other tests it is also applicable to ordinal data. The Tau-U summary index Tau-U_A vs. B − trend A_ can be understood as an effect size coefficient, showing the proportion of the data that improves from baseline to intervention after adjusting for the baseline trend [54, 55]. Follow-up phase data was not included in the Tau-U calculation. The magnitude of Tau-U statistics were interpreted similarly to correlation and effect size statistics: 0.00–0.25 (very low), 0.26–0.49 (low), 0.50– 0.69 (moderate), 0.70–0.89 (high) and 0.90–1.00 (very high) [56]. Tau-U_A vs B − trend A_ summary indices were calculated with RStudio version 1.2.5042 [57] running R 4.0.0 with Rtools40 installed [58], using the packages SingleCaseES version 0.4.3 [59] and readxl version 1.3.1 [60]. See Additional file 2 for the R script and Additional file 3 for the excel file which was loaded into R.

## Results

The demographic and clinical characteristics of the participants along with number of assessments, training sessions and training time are shown in Table 1. The recruited participants hailed from different ethnicities and had an educational background spanning less than 9 years education to completed third cycle education, with the most common background being Swedish ethnicity and more than 12 years of education. According to the medical records, one participant had some residual perceptual and cognitive deficits, although these deficits were relatively mild and did not interfere with co-operation during testing or intervention. None of the participants had documented neglect, and two participants had some residual communication difficulties (slower in speaking). All participants completed the baseline assessments and were followed-up 6 months post intervention.

Training time was mostly limited by the participants’ time and energy, and not by limitations in system accessibility or capacity. Three participants (P1, P3, P5) trained approximately 3 times per week and therefore reached the highest training dose of 739–915 min in total. See Additional file 4 for further details on the training sessions. Three participants (P2, P4, P6) attended fewer sessions due to practical reasons such difficulties arranging transport or having a busy schedule. One participant (P7) was interrupted from training after 4 training sessions due to a hip fracture after a fall at home. This was also the reason for missing data points on assessments during the intervention phase in P7. For this reason, the P7 was excluded from the visual analysis.

No serious adverse effects were observed during or after training. One participant felt slightly unsteady for a few hours post-training after the first training sessions. Another participant perceived standing on a virtual platform in the Beat Saber game as somewhat intimidating. The median physical exertion after a training was graded by participants between 12 and 18 (Table 1).

Participants were overwhelmingly positive when asked about their thoughts on the training session; all participants expressed that training was “good” and/or “fun” during the intervention phase. They often commented the improvement they saw in their in-game performance. Participants described a positive feeling of being in another world in which they could move more easily. Other improvements that they mentioned were for upper extremity functioning (P4), pain (P4), spasticity (P3), neglect (P7) and walking (P6). P1 explicitly said he did not perceive any improvement outside of the game. The attending researcher noted that two participants (P1 and P3) became independent in using the VR system.

Two participants had undergone a 3-weeks intensive rehabilitation between the last intervention and 6-monts follow-up assessment (P2 and P6), and one (P2) had participated in another 6-weeks interventional study targeting upper extremity function. Other participants did not report any changes in their rehabilitation. None of the participants had changed their overall physical activity level as measured with SGPALS.

**Fig. 3:**
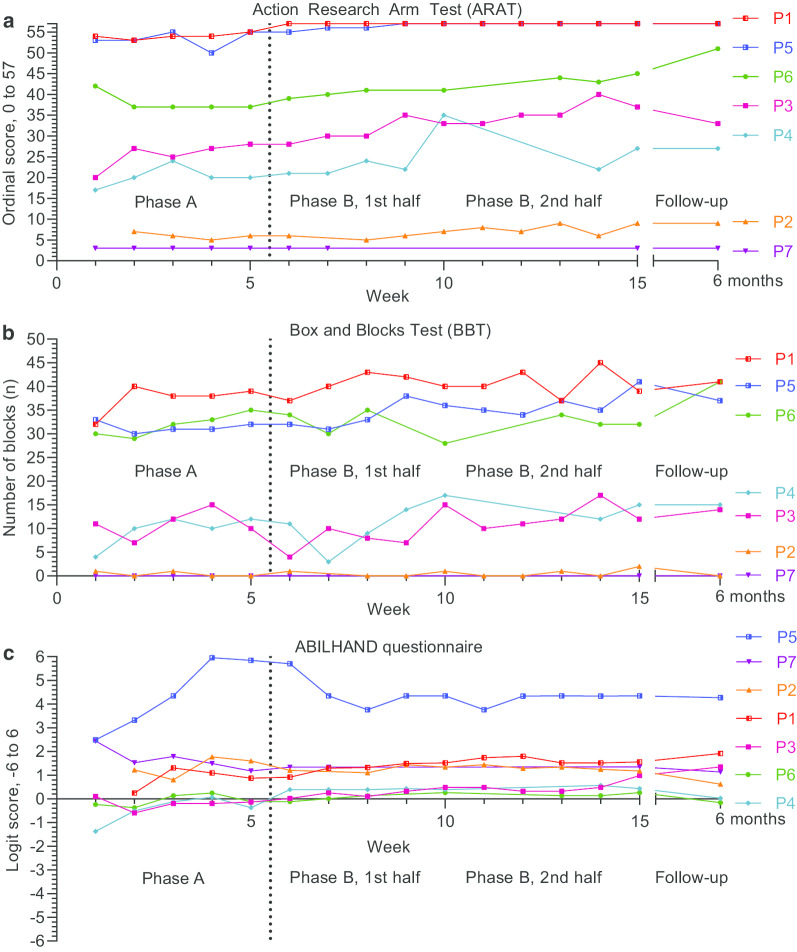
Activity domain outcome measures: Action Research Arm Test (**a**), Box and Blocks Test (**b**) and the ABILHAND (**c**) questionnaire. Outcomes were assessed on a weekly basis during baseline (Phase A) and intervention (Phase B), and at 6-month follow-up

**Fig. 4:**
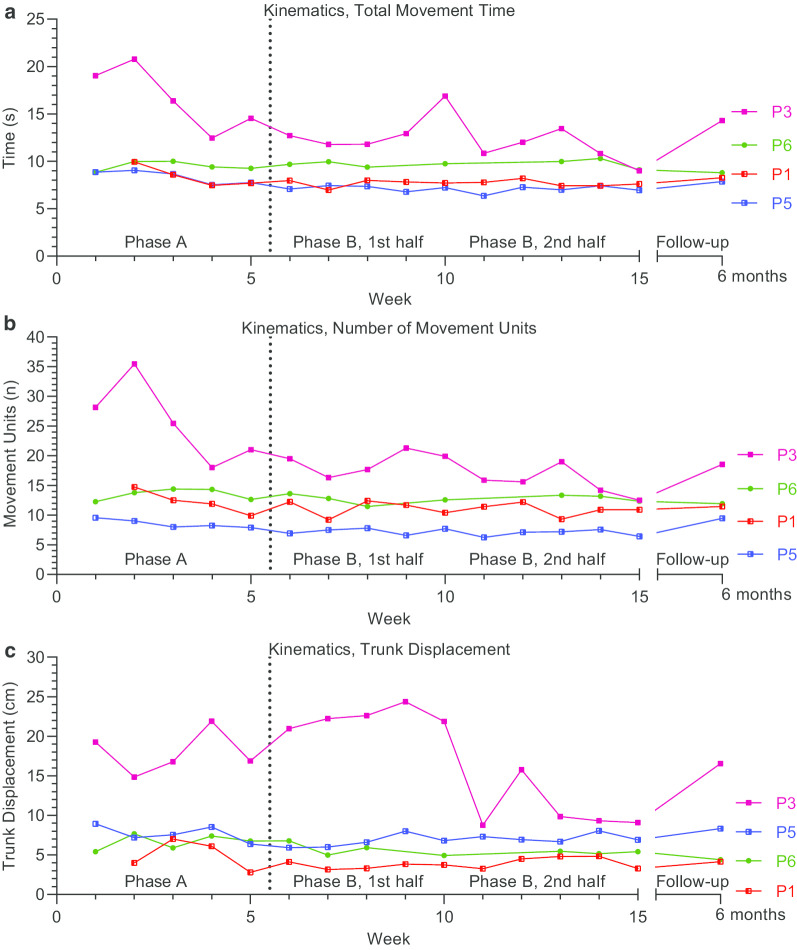
Kinematic body function outcome measures: Total Movement Time (**a**), Number of Movement Units (**b**) and Trunk Displacement (**c**). Outcomes were assessed on a weekly basis during baseline (Phase A) and intervention (Phase B), and at 6-month follow-up

Outcomes were plotted on an outcome basis, with activity domain outcomes in Fig. 3 and body function domain outcomes in Fig. 4. Outcomes plotted on an individual basis with additional annotation is available in Additional file 5.

### Activity domain outcome measures

#### Action Research Arm Test

Regarding baseline stability and trend, visual analysis indicated a relatively stable baseline for all six participants (Fig. 3a), although in two participants (P3 and P4) a positive trend which plateaus in the end of the baseline could be observed. In phase B, trends remain positive for all participants except P1 and P5 who reached the ceiling effect early the intervention phase. All six showed increased levels during the phase B. This level shift was in particular visible in the second half of the phase B. Three participants (P1, P3, P5) had little to no overlap between phase A and B, while P2, P4, P6 had some overlap.

The Tau-U scores for ARAT were compatible with the improved levels, as well as the relatively unchanged trends from baseline to intervention found in the visual analysis (Table 2). Improvements detected during intervention phase compared to baseline after adjusting for the baseline trend was large or very large (Tau-U > 0.70) in four participants, and moderate (Tau-U > 0.50) in two participants. The first high score in P6, resulted in an increased overlap between phases in the visual analysis, but due to lower following scores at baseline, the Tau-U still showed large effect in this participant. In three participants (P3, P4, and P6) the ARAT scores were large than the MCID of 5.7 points in the end of the intervention phase when compared to the last baseline score. In two participants (P1 and P5) a ceiling effect was present in ARAT, and improvement beyond the MCID could not be detected.

**Table 2:** Tau-U summary index for clinical and kinematic outcome measures

Outcome measures	Participants						
	P1	P2	P3	P4	P5	P6	P7
Activity capacity assessments							
Action Research Arm Test	*0.90*	*0.50*	*0.84*	*0.66*	*0.92*	*0.89*	0.00
Box and Block Test	*0.50*	0.18	− 0.10	0.14	*0.78*	− 0.14	0.00
ABILHAND	*0.85*	− 0.22	*0.92*	*0.83*	− 0.24	0.37	− 0.07
Kinematic measures of drinking task							
Total movement time	0.25	–	*0.64*	–	*0.88*	− 0.17	–
Smoothness. number of movement units	0.30	–	*0.68*	–	*0.84*	0.49	–
Trunk displacement	0.25	–	0.08	–	0.40	*0.66*	–

Tau-U index: 0.00–0.25 very low, 0.26–0.49 low, 0.50– 0.69 moderate, 0.70–0.89 large, 0.90–1.00 very large effect. Negative numbers indicate a negative effect. Tau-U ≥ 0.50 is indicated in italics

At 6 months follow-up the ARAT scores showed further improvement in one participant (P6), while in all others the ARAT remained slightly higher than the scores at baseline (Fig. 3a).

#### Box and Blocks Test

Visual analysis showed fair baseline stability. Baseline trends were either neutral (P2, P3) or plateaued (P1, P5, P4), although P6 had a continuously positive baseline trend (Fig. 3b). P5 showed a clear positive trend during the intervention. While overall variability was high for most participants in both phases, the greatest variability could be seen for P3 in the baseline, which then decreased throughout the intervention. Levels increased between baseline and the second half of the intervention for 3 participants (P1, P4 and P5).

This level shift with low overlap was supported by the moderate to large Tau-U scores for P1 and P5, respectively (Table 2). The very low Tau-U for P4 could be explained by the plateauing baseline trend, as well as the low scores in the first 3 weeks of the intervention. Only one participant (P5) showed a change large than the MDC of 5.5 cubes in the end of the intervention phase when compared to the last baseline score.

Follow-up scores were analogous to the latter half of the intervention, except for P6 who scored substantially higher. P6 had limited training during the intervention (198 min, Table 1) yet participated in 3 weeks intensive rehabilitation prior to the follow-up assessment.

#### ABILHAND questionnaire

Visual analysis exhibited plateauing baselines around 0 logits (P3, P4, P6), 1.25 logits (P1, P2), or close to the ceiling effect of 6 logits (P5) (Fig. 3c). Interventional trends were on a whole neutral, although P3 and P1 presented a positive trend in the first and in the second half of the intervention phase, respectively. There were little to no interphase overlap in phase B for P1, P3 and P4. Notably, interindividual comparative scores for the self-perceived manual ability measured by ABILHAND differed from all other performance-based outcomes, which shared more consistent patterns. For example, P2, who showed the lowest motor function in performance tests scored on par with or higher than most other participants in ABILHAND.

The low overlap with increased levels as manifested with the visual analysis for 3 participants were supported by the large (P1 and P4) and very large (P3) Tau-U scores (Table 2). In four participants (P1, P3, P4, and P6) the ABILHAND logits were large than the MCID of 0.35 logits in the end of the intervention phase when compared to the last baseline score.

The 6-month follow-up revealed decreased ABILHAND logits compared to the interventional phase scores for all participants except P1 and P3.

#### Body functions domain outcome measures—kinematics

Four participants (P1, P3, P5, P6) with sufficient motor function were able to perform the kinematic drinking task with the more-affected arm (Fig. 4). A clear negative and thus improving trend in movement time and movement units was seen with visual analysis during the baseline for P3, while this trend was less pronounced in P1 and P5. During the intervention phase, the levels improved, i.e. were lower, and there was a little to no interphase overlap in P3 and P5 concerning movement time (Fig. 4a) and movement units (Fig. 4b). The visual analysis showed a high degree of variability in P3 throughout both phases for all 3 kinematic variables, while it was more modest for the other participants. P3 also showed improved levels in all three kinematic measures, mainly in the second half of the intervention phase. These observed improvements in P3 were beyond the established clinically important difference of 2.4 s, 3.3 movement units and 2.0 cm for the movement time, number of movement units and trunk displacement, respectively [43]. A small improvement in trunk displacement was noted for the P6 in the intervention phase (Fig. 4 C).

The improved levels seen in the visual analysis were supported by Tau-U in all cases, showing moderate to large effect (Tau-U 0.66 to 0.88), except for the late improvement observed in P3 in trunk displacement that showed very low effect (Tau-U 0.08) (Table 2).

In summary, two out of four participants (P3 and P5) tested with kinematics improved in terms of movement time and movement units, as substantiated with both visual and statistical analysis. Two out of four (P3 and P6) improved in trunk displacement, in which the late improvements in trunk displacement in P3 were not supported by Tau-U analysis.

After 6 months, the observed improvements showed, however, some reversion in most of the participants. The 6 months follow-up measures were akin to their respective final two baseline measurements for all participants except P6, who achieved personal bests.

#### Additional clinical assessments

Scores from the additional clinical assessments of sensorimotor function assessed pre- and post-intervention as well as at 6 months follow-up show relatively stable values over the course of the study (Table 3). The change in FMA-UE scores between pre- and post-intervention varied between 0–5 points.

**Table 3:** Scores of the additional clinical assessments prior and after intervention as well as at 6-months follow-up

ID	FMA-UE (0–66)			Sensation (0–12)			ROM (0–24)			Pain (0–24)			Spasticity (0–20)		
	A	B	FU	A	B	FU	A	B	FU	A	B	FU	A	B	FU
P1	63	66	64	12	12	12	24	24	24	24	24	24	0	0	0
P2	18	17	22	10	11	12	21	20	21	22	23	23	7	9	5
P3	42	46	45	10	12	11	19	22	24	18	23	18	4	4	4
P4	35	40	39	12	12	12	22	22	22	23	21	21	2	3	3
P5	51	56	52	12	12	12	19	22	21	21	23	23	5	5	3
P6	44	49	47	12	12	12	21	21	22	23	22	24	4	3	1
P7	15	15	15	8	7	7	21	21	23	23	23	23	9	10	8

*FMA-UE* Fugl-Meyer Assessment—Upper Extremity, *ROM* range of motion, *A* phase A (baseline), *B* phase B (post intervention), *FU* 6 months follow-up

## Discussion

This study evaluated the potential effects of commercial head-mounted virtual reality on upper extremity functioning in individuals with chronic stroke. Acceptable adherence to the training protocol was reached in 6 out of 7 participants and no serious adverse effects were observed during or after the intervention. The most played VR game was a rhythm-based game that did not require any manipulation of the buttons by the player and allowed a variety of adjustments in terms of speed, gaming difficulty and other personal preferences. Thus, most of the training was focused on the arm and hand movements, and less on the finger movements. A positive trend of improvement, at least in one outcome measure, was observed in all 6 participants adhering to the training protocol. Independent of the impairment level, all 6 participants showed improvements in upper extremity activity capacity, assessed with ARAT. The 3 participants who had the highest training dose (739–915 active training minutes) showed improvements in 3 to 5 outcome measures out of total 6. These findings indicate that commercial HMD VR might be a useful tool for people with chronic stroke to improve their upper extremity functioning.

### Who might benefit most from VR training?

Results indicated noticeable improvements in five participants (P1, P3, P4, P5, P6). One participant (P2) with poor initial motor function did reach the cut-off value indicating a moderate effect in statistical analysis for ARAT, although this finding was less pronounced in the visual analysis. One participant (P7), who needed to interrupt the intervention premature due to a fall at home did not show any change. Interestingly, the group who responded better to the intervention had baseline ARAT and FMA-UE scores above 15 and 30 points, respectively, in contrast to P2 who had ARAT below 10 and FMA-UE 20 points. Thus possibly, the VR intervention, as used in this study, might have the best benefit for those with moderate to mild upper extremity impairment. Similar to our results, the non-immersive VR based gaming demonstrated positive effects primary in those with moderate to mild upper extremity impairment [6, 16]. Thus, possible effects of VR gaming, immersive or not, in people with poor motor function after stroke remain unknown.

As for the dose, three participants (P1, P3 and P5) with the highest training dose showed improvements in several outcomes. Nevertheless, even participants with lower dose showed improvements in some outcome measures. The only exception was the participant with severe motor impairment (P2) and the participant who interrupted the study. A training time around 500 min, as used in a large randomized control trial using video-based non-immersive VR gaming (Nintendo Wii) as add-on therapy, showed significant positive effects on activity performance time in subacute stroke [16]. An appropriate dose for HMD VR is largely unknown, although based on our data, a time between 200 and 900 min had some effect for upper extremity functioning in people with chronic stroke. However, training levels of 900 min, which means at least 30 min of VR training 3 times per week for 10 weeks, might have a higher potential effect on upper extremity functioning.

### What outcomes should be used?

The VR training, as used in this study, was primarily expected to target the activity domain of functioning. The gaming is a task- and performance-oriented exercise, which do not emphasize the quality of movement nor whether the task is accomplished by using a certain muscle group or movement. Accordingly, in the current study, the training effect was most evident in the activity domain as assessed by the ARAT. Thereby, ARAT, as an activity level assessment could be recommended as a first-choice outcome for similar interventional studies. Activity level outcome, the Wolf Motor Function Test, was used as primary endpoint in a large randomized control trial using video-based non-immersive VR gaming [16]. The task execution time was significantly improved after VR intervention, although this result was not superior to the same dose recreational activity intervention [16].

In the current study, we also included kinematic movement analysis of the drinking task to objectively evaluate the changes in movement performance and quality. Since this task necessitates an ability to grasp the glass and drink a sip water with the more-affected arm, only 4 out of 7 participants were able to perform the task. After VR training, two participants showed consistently shorter movement times accompanied by improved movement smoothness; and in two, the compensatory forward movement of trunk during task execution was decreased. However, improvements larger than the established clinically important difference were only present for all three kinematics (movement time, smoothness and trunk displacement) in one participant (P3) [43]. Kinematics have previously only sparsely used to evaluate the effects of VR gaming and might therefore provide new insights on possible effects on motor performance and quality [61, 62]. While a shorter movement time reported in this study echoed results from previous research [16] which showed improvements in task execution time after video-based VR intervention, improvements in movement smoothness are novel. These results are promising and show that some improvements might as well be expected in the domain of motor function when measured objectively with kinematic analysis.

As a fast, mobile and straight-forward test yielding data on a discrete integer scale as opposed to an ordinal scale, BBT is appropriate as a composite measure for speed and precision of finger, hand and arm function [32]. Limitations of this outcome became, however, visible in the present study. A relatively large variability coupled with the seemingly low responsiveness seen in the data may have compromised the statistical power. A higher floor effect of BBT than ARAT was also observed for P2.

While there seemed to be a high degree of agreement of inter-individual relative scores between outcomes, the self-perceived manual ability in daily activities as assessed by ABILHAND, displayed incongruent patterns. Discrepancies between observed and self-reported measures are not uncommon and can partly be explained by inter-personal differences in perception of problems and how these affect the daily life activities. In chronic stroke, one in five showed discrepancies between self-reported and observed upper extremity outcomes [63]. With this background, the self-reported outcome measures are often advocated to be included in clinical studies in order to cover and better understand patient perspectives [63–65].

### How did the VR gaming work?

Players with severe upper extremity impairment could play Beat Saber on HTC Vive, albeit by using Velcro straps to retain the grasp of the hand control. To overcome this limitation, recently available lighter and more ergonomic hand controls with built-in straps, such as Valve Index, could be considered. Finger tracking independent of hand controls, such as Leap Motion [66], represent another opportunity for participant interaction when the technology has been developed further. Meanwhile, if Beat Saber would be the sole available game appropriate for the intervention then the stand-alone and thus much more affordable Oculus Quest could be used instead.

The median physical exertion after training, as reported by the participants, ranged between 12 and 18. This corresponds to somewhat hard to very hard perceived exertion, on the Borg RPE scale. Similar exertion rates have been reported previously in intervention studies using video-based non-immersive VR gaming in stroke [16, 67]. Even when the focus of this study was not on cardiovascular health, previous research using VR-based gaming have shown positive effects to fitness and physical activity [68]. Taken the low levels of physical activity often described in people with stroke, playing engaging VR games might be beneficial from an exercise point of view [69].

Screening 300 of the approximately 5000 titles available for HTC Vive primarily yielded 1 game. This indicate that while the assortment of commercial HMD VR games is staggering, the amount of games appropriate for upper extremity rehabilitation post-stroke is miniscule. Other HMD VR rehabilitation studies using commercial hardware use custom rehabilitation software, often developed by the researchers themselves [19, 20, 70, 71]. It seems, however, that the custom hardware has limited production values and weren’t developed with the help of commercial game developers in order to maximize intrinsic gameplay rewards, i.e. making the game itself as fun as possible in order to boost adherence and the exploitation of reward mechanisms. Reaching out to successful game developers in order to modify existing commercial games or to develop novel ones with rehabilitation in mind seem to be untested.

### Strength and limitations

With 7 participants and 6 outcome measures, this represented a large single case design study. Furthermore, the recruited participants were a heterogeneous group, which is a strength for single case design. The results from the current study can guide researchers to select suitable study design, what outcome(s) to assess, what participants to recruit and how many are needed when designing larger studies. An appropriate next step could be a phase 2 VR study with focus on feasibility, acceptability and initial clinical efficacy [10]. The open commercial nature of both the hardware and software used in this study facilitate adoption in wider research community, both in terms of ease of access and cost.

Like a few previous rehabilitation studies [64, 65], the customary visual analysis was complemented with Tau-U [53], which strengthens the findings. Baseline Corrected Tau (BC-Tau) was another possibility [63], however the baseline correction of BC-Tau was strict and depended on only adjusting for statistically significant baseline trends, which were an issue since Monte Carlo simulations for BC-Tau indicated exceptionally low power for baselines consisting of 5 measurements [63]. A strength BC-Tau would have over Tau-U if it could appropriately adjust for baseline trend in our data, is that unlike Tau-U, BC-Tau yields further statistics such as p-values.

A downside of the intervention, at least in the context of this study, was the requirement of having a researcher, experienced with both rehabilitation and VR gaming, on site for all training sessions. Furthermore, the single case design was not experimental and thus did not permit causal inference. A common motif in baseline measurements was the initial improvement that plateaued towards end of the phase. While the relative stability of plateaued values was acceptable for comparing level changes between phases in our single case design, similar baseline changes would make it difficult to establish causality [27, 49]. Considering these baseline changes as well as the slow improvement that one would expect from a successful chronic stroke rehabilitation intervention, an experimental single case design might not be appropriate for a next phase study which attempt to determine the interventions effectiveness. The baseline improvements seen in this study might partially have caused by familiarization and training effects in some of the participants due to the repetitive scheme of the assessments. In some participants, the baseline assessments might as well be acting as somewhat of a rehabilitation activity by themselves. The heterogeneity of the participants extended to upper extremity function, so much so that floor and ceiling effects of several outcomes came into play, masking potential changes. Due to the study design, the results cannot be generalized to the stroke population at large and need to be interpreted individually.

## Conclusions

The results generated in this study indicate that off-the-shelf, room-scale head-mounted-display VR has potential for upper extremity rehabilitation in individuals with chronic stroke. The selection of available games appropriate for stroke population is, however, limited. Taken the promising results along with experiences and lessons learned, phase 2 studies evaluating feasibility and initial efficacy are warranted. Future studies may want to aim for 200–900 min total training time, perhaps toward the upper portion of the range. In the long run, these future studies may enable the addition of another fun and cost-effective tool in the arsenal of rehabilitation health-care professionals.

## Supplementary information

**Additional file 1.** Describes the screening and testing process for the games.

**Additional file 2.** The R script which was used to calculate the Tau-U_A vs. B − trend A_ summary index for the primary outcomes.

**Additional file 3.** The excel file with primary outcomes which was imported to R for the Tau-U analysis.

**Additional file 4.** This excel file contains all data which was generated with or analyzed in this study. It is divided into 3 tabs: Primary outcomes, Additional assessments, and Training sessions.

**Additional file 5.** Outcomes plotted on an individual level. The dotted lines are linear models fit to the corresponding phase. The area shaded grey around the fit line is the 95% confidence interval for the model. The horizontal black lines are the medians for Phase A, 1st, and 2nd half of phase B respectively. The vertical bar between phase A and B is the MCD (BBT) or MCID (all other outcomes) for the outcome, anchored at the phase A median.

## Data Availability

All data generated or analyzed during this study is included in this published article and its additional files (Additional file 4).

## Abbreviations

VR: Virtual reality
HMD: Head-mounted display
ARAT: Action Research Arm Test
BBT: Box and blocks test
FMA-UE: Fugl-Meyer Assessment of Upper Extremity
mAS: Modified Ashworth Scale
SGPALS: Saltin-Grimby Physical Activity Level Scale
ICF: International Classification of Functioning, Disability and Health
MCID: Minimally clinically important difference
MDC: Minimally detectable change
