# Supplementary material for: Commercial head-mounted display virtual reality for upper extremity rehabilitation in chronic stroke: a single-case design study
Source: J Neuroeng Rehabil. 2020 Nov 23;17:154. doi: 10.1186/s12984-020-00788-x (PMC7686731; doi:10.1186/s12984-020-00788-x)
Supplement: Supplementary file 1 — Additional file 1. Describes the screening and testing process for the games. [file 12984_2020_788_MOESM1_ESM.docx]

# Additional file 1 – The screening process and downloaded games

## Screening process

While there are thousands of available HMD VR games available, only a handful might be appropriate for specific use-cases such as upper extremity rehabilitation for individuals with stroke. In this study, the following criteria were considered to determine its potential suitability for this use-case:

### Criteria

- Upper extremity interaction.
  The game needs to force or at least highly encourage the player to use the affected arm, but many games do not. For example, games focused on exploration, puzzles and stories can typically be played perfectly fine without using the affected arm. Ideally, the focus of the game itself should be the movement of both arms.
- Difficulty scaling.
  The game should be of an appropriate difficulty level for the user no matter how proficient they are. Thus, one should be able to fine-tune the difficulty within a massive range so that people with severely impaired upper extremity function without any video game experience can overcome an appropriate challenge, just as much as someone with a milder upper extremity impairment who has extensive video game experience.
- Cyber sickness.

Modern HMD VR hardware and most software is designed to minimize cyber sickness. While there are few issues with the hardware side, there are quite a few games which generates a noticeable amount of cyber sickness. As some users are more sensitive than others, this is a major concern for widespread usage.

- Ease of use.
  It is undesirable if the user needs to be a proficient VR gamer to be able to use the software. Game design meant to be cognitively challenging, while potentially fun, distracts from focused upper extremity rehabilitation, or even disallows it completely if too challenging for the user. Likewise, a badly designed user interface presents an obstacle.
- Fun factor.

The intrinsic reward of playing the game itself is integral to this rehabilitation.

- Production quality.

A substantial proportion of available games is worthless “shovelware”. Even games created by more serious developers varies in quality, partially due to the video game industry trend of publishing games as “early access” before they are finished.

There is a high degree of correlation between factors. For example, a shovelware game is likely to be boring, have an atrocious user interface, generate massive amounts of cyber sickness, have inflexible difficulty scaling and not be particularly interactive.

### Procedure

1. Filter games on the Steam platform to only show VR-only, single player games available for HTC Vive. Games not designed for VR and merely ported from other platforms are probably less likely to be appropriate for our purposes. The same can be said about games with a multiplayer focus.
2. Sort from highest to lowest popularity.
3. Browse the following categories one by one:
   1. All games (no further narrowing)
   2. Action
   3. Casual
   4. Sports
4. For each game browsed, do the following. Move on to the next game if it is deemed inappropriate at any step:
   1. Read the written description.
   2. Watch the gameplay videos on the steam store page.
   3. Read a handful of steam user reviews.
   4. Watch a handful of gameplay videos on youtube.
   5. Read a handful of written reviews.
   6. Play demo version, if available.
   7. Purchase the game and test it.

## Downloaded games

Most games were deemed obviously inappropriate after just reading the written description. After reviewing approximately 300 games, the following 6 games were downloaded:

- **The Lab.** This game has a selection of mini games, of which the archery game was the most promising and saw some limited use.
- **NVIDIA VR Funhouse.** This game has a selection of mini games, of which the boxing, balloon-popping and shooting gallery games were the most promising and popular among the participants. It was, however, marred by recurring game-breaking bugs which are only bound to get worse as the game is no longer updated.
- **Pierhead Arcade.** This game has a selection of mini games, based on analogue arcade games. It did not generate interest among the participants and compared to the other games it did not engage the upper extremities to the same degree as them.
- **Biathlon Battle VR.** We did not offer this biathlon game to participants, as the cyber sickness and risk of falling generated from sudden stops at high in-game speeds was deemed unacceptable.
- **Climbey.** A climbing game where you need to use both hands. Greatest potential downside was the cybersickness generated from jumping or falling. Only the demo version was played. Participants were offered to try the demo but were not interested, so it was never purchased in full.
- **Beat Saber.** The main game played in this study. In Beat Saber, the user has one lightsaber in each hand, which is used to cut blocks to the sound of music. The gameplay forces the player to use both hands. The difficulty can be adjusted in a massive range, which stretches from beyond the best of human capacity to a negligible difficulty level. The difficulty level can be fine-tuned with a great variety of variables which include song (how difficult each song is varies), the song difficulty level (typically 5 levels, with different amount of blocks, block speed and complexity of patterns), song speed, disabling block orientation (so that they can be cut in any direction), making blocks more difficult to see and changing the defeat condition (from impossible to lose, to losing if a single block is missed). There is a risk of sensory overload even at the lowest difficulties, although lowering the volume and enabling options reducing special effects can somewhat mitigate this. The gameplay itself do not require the user to press buttons, allowing individuals with severe upper extremity impairment to play it e.g. through attachment of the hand controller to the hand with Velcro straps. It was designed to be played standing but can be played sitting.
