# Supplementary material for: Commercial head-mounted display virtual reality for upper extremity rehabilitation in chronic stroke: a single-case design study
Source: J Neuroeng Rehabil. 2020 Nov 23;17:154. doi: 10.1186/s12984-020-00788-x (PMC7686731; doi:10.1186/s12984-020-00788-x)
Supplement: Supplementary file 5 — Additional file 5. Outcomes plotted on an individual level. The dotted lines are linear models fit to the corresponding phase. The area shaded grey around the fit line is the 95% confidence interval for the model. The horizontal black lines are the medians for Phase A, 1st, and 2nd half of phase B respectively. The vertical bar between phase A and B is the MCD (BBT) or MCID (all other outcomes) for the outcome, anchored at the phase A median. [file 12984_2020_788_MOESM5_ESM.pdf]

P1 ARAT

ARAT MCID = 5.7

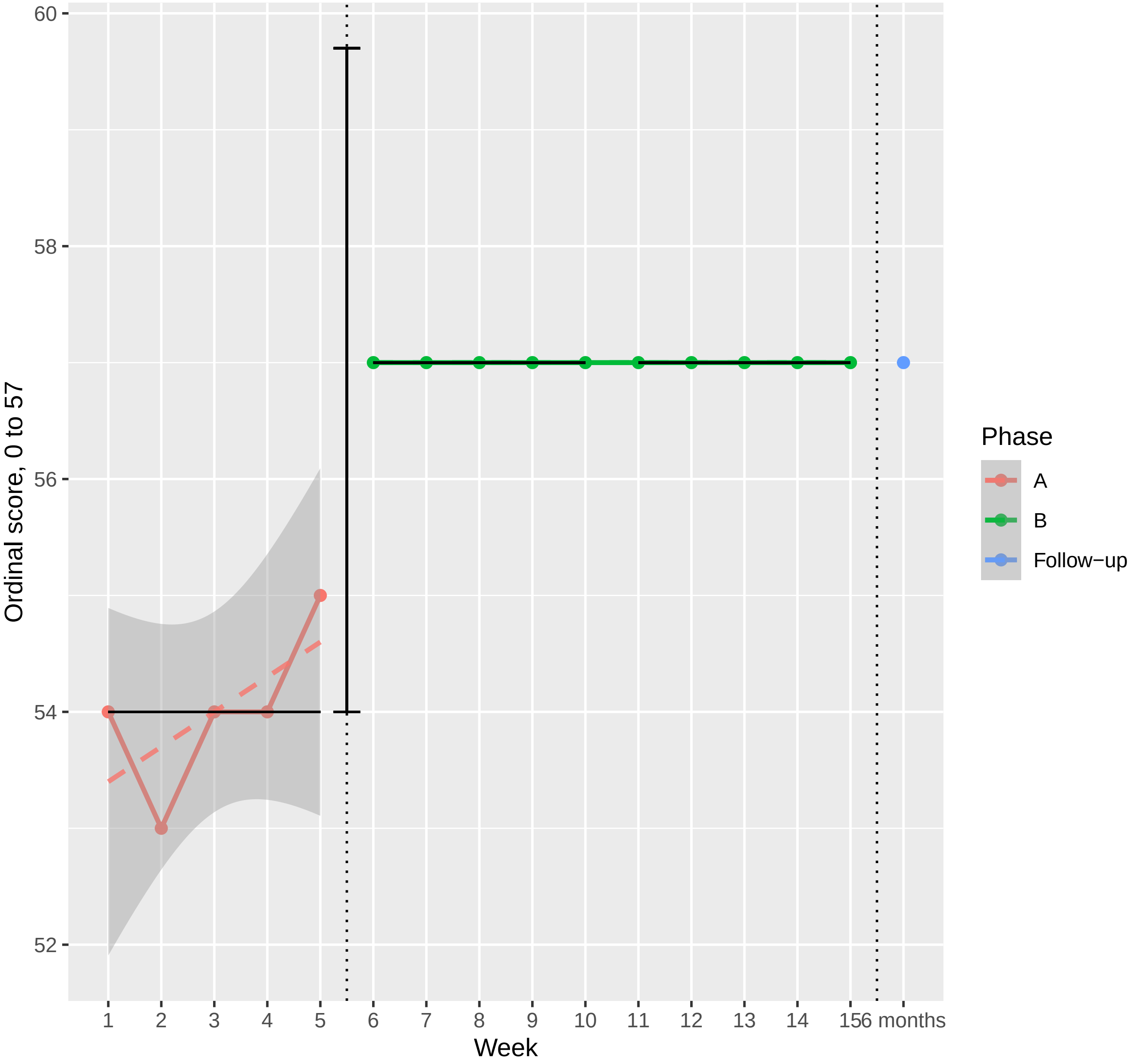

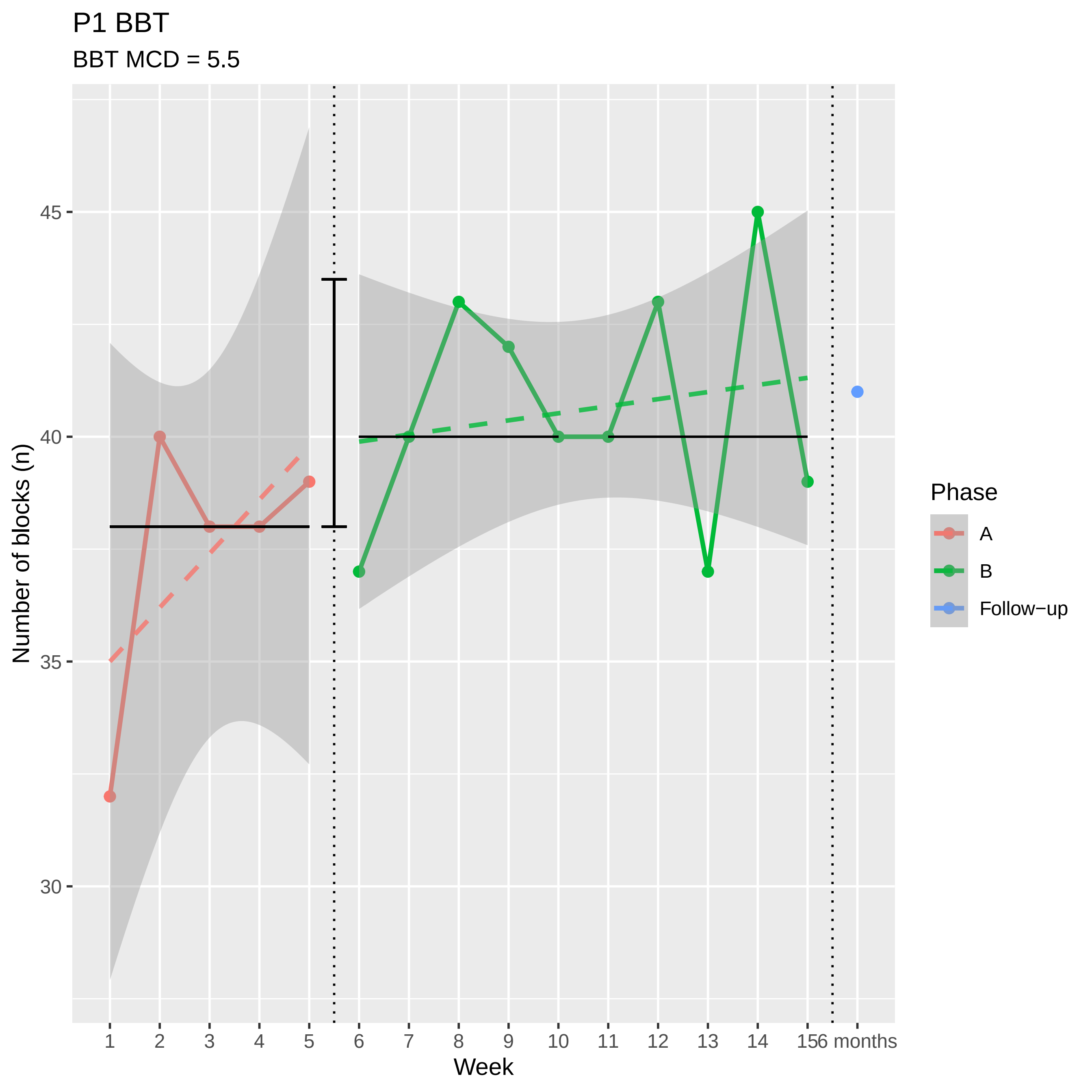
P1 BBT
BBT MCD = 5.5
Number of blocks (n)
45
40
35
30
Week
1
2
3
4
5
6
7
8
9
10
11
12
13
14
15
6 months
Phase
A
B
Follow−up

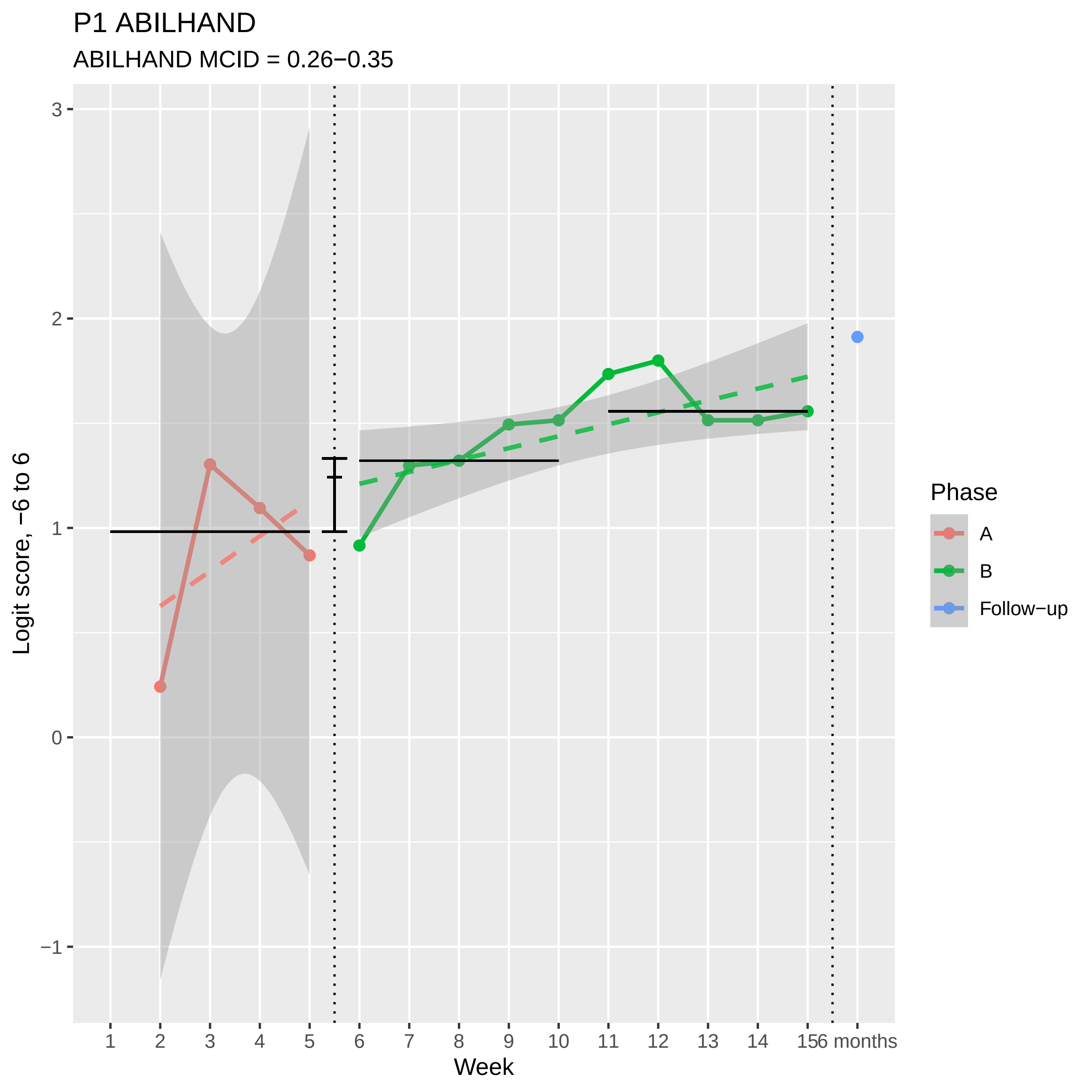

P1 ABILHAND
ABILHAND MCID = 0.26−0.35
Logit score, −6 to 6
3
2
1
0
−1
1
2
3
4
5
6
7
8
9
10
11
12
13
14
15
6 months
Week
Phase
A
B
Follow−up

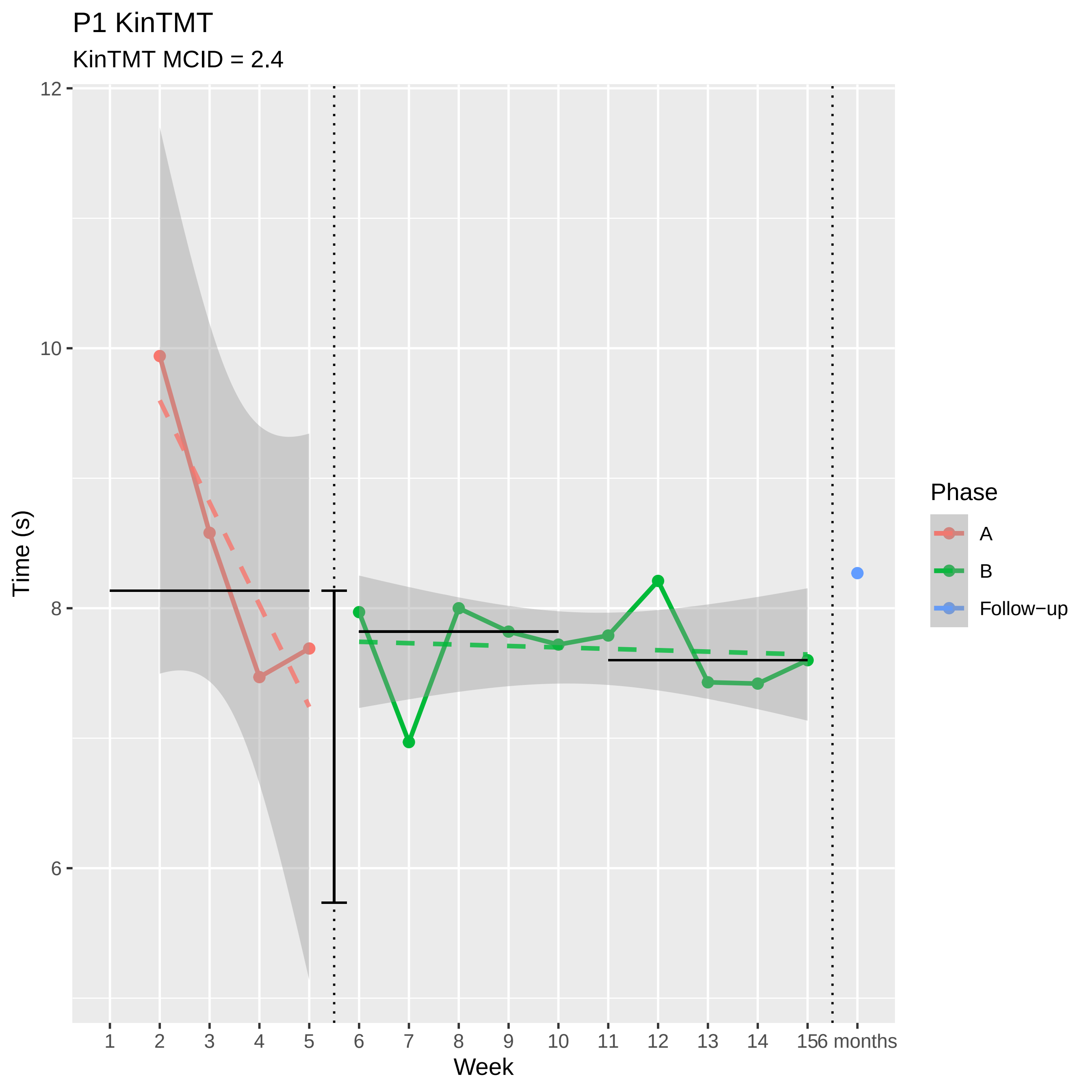
P1 KinTMT
KinTMT MCID = 2.4
12
10
8
6
Time (s)
1
2
3
4
5
6
7
8
9
10
11
12
13
14
15
6 months
Week
Phase
A
B
Follow−up

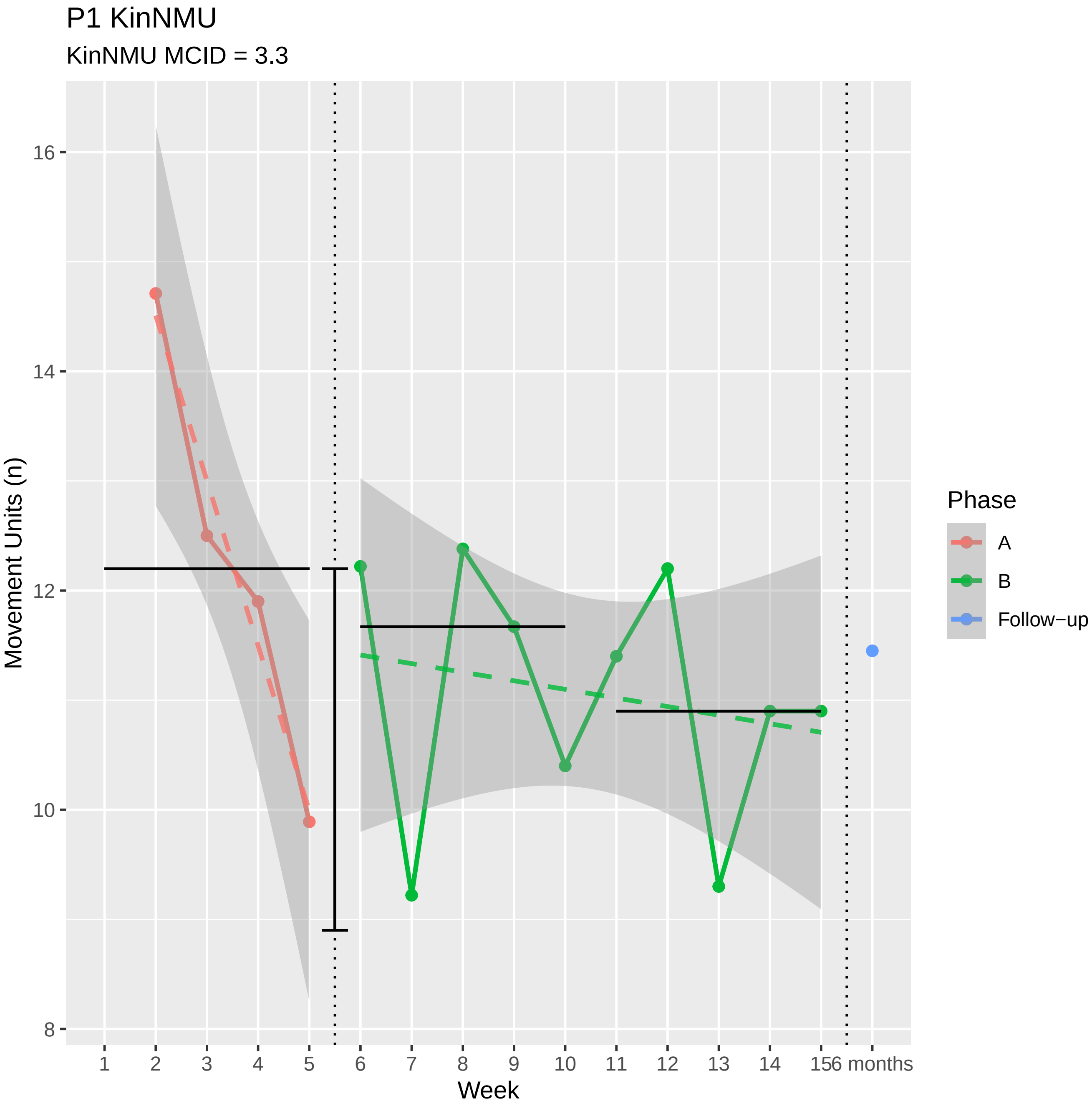
P1 KinNMU
KinNMU MCID = 3.3
Movement Units (n)
16
14
12
10
8
1
2
3
4
5
6
7
8
9
10
11
12
13
14
15
6 months
Week
Phase
A
B
Follow−up

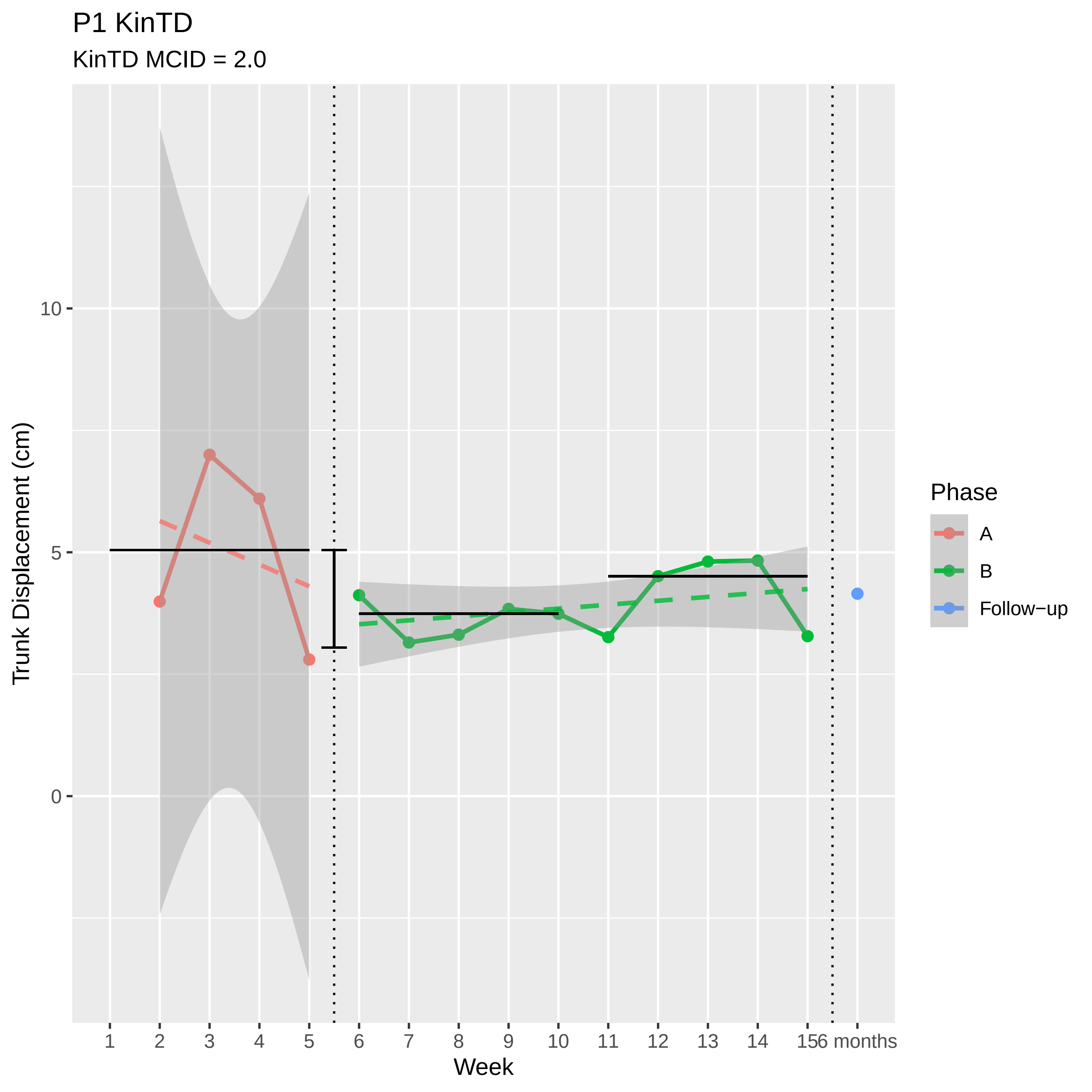
P1 KinTD
KinTD MCID = 2.0
Trunk Displacement (cm)
10
5
0
1
2
3
4
5
6
7
8
9
10
11
12
13
14
15
6 months
Week
Phase
A
B
Follow−up

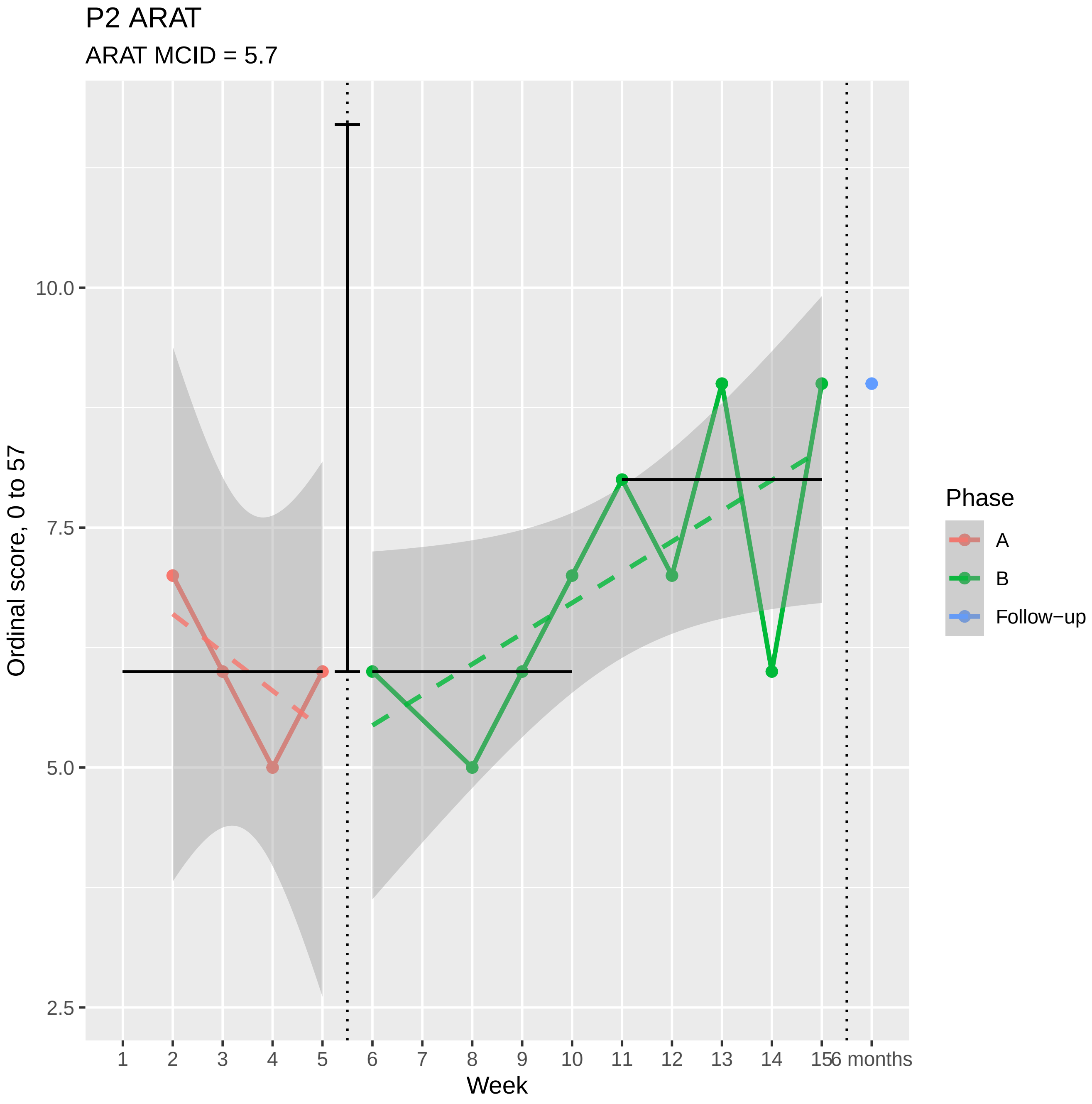

P2 ARAT
ARAT MCID = 5.7
Ordinal score, 0 to 57
10.0
7.5
5.0
2.5
1
2
3
4
5
6
7
8
9
10
11
12
13
14
15
6 months
Week
Phase
A
B
Follow−up

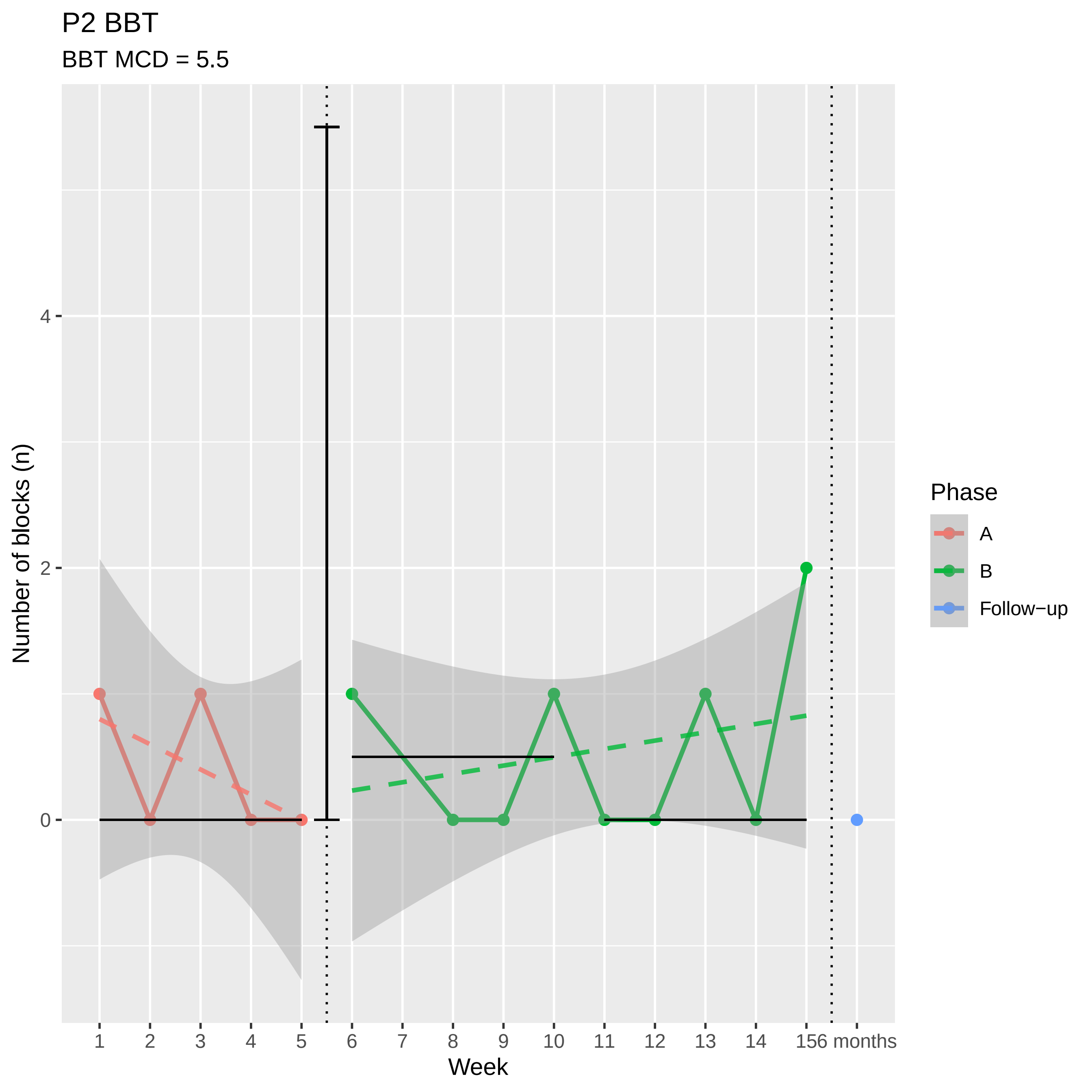
P2 BBT
BBT MCD = 5.5
Number of blocks (n)
0
2
4
1
2
3
4
5
6
7
8
9
10
11
12
13
14
15
6 months
Week
Phase
A
B
Follow−up

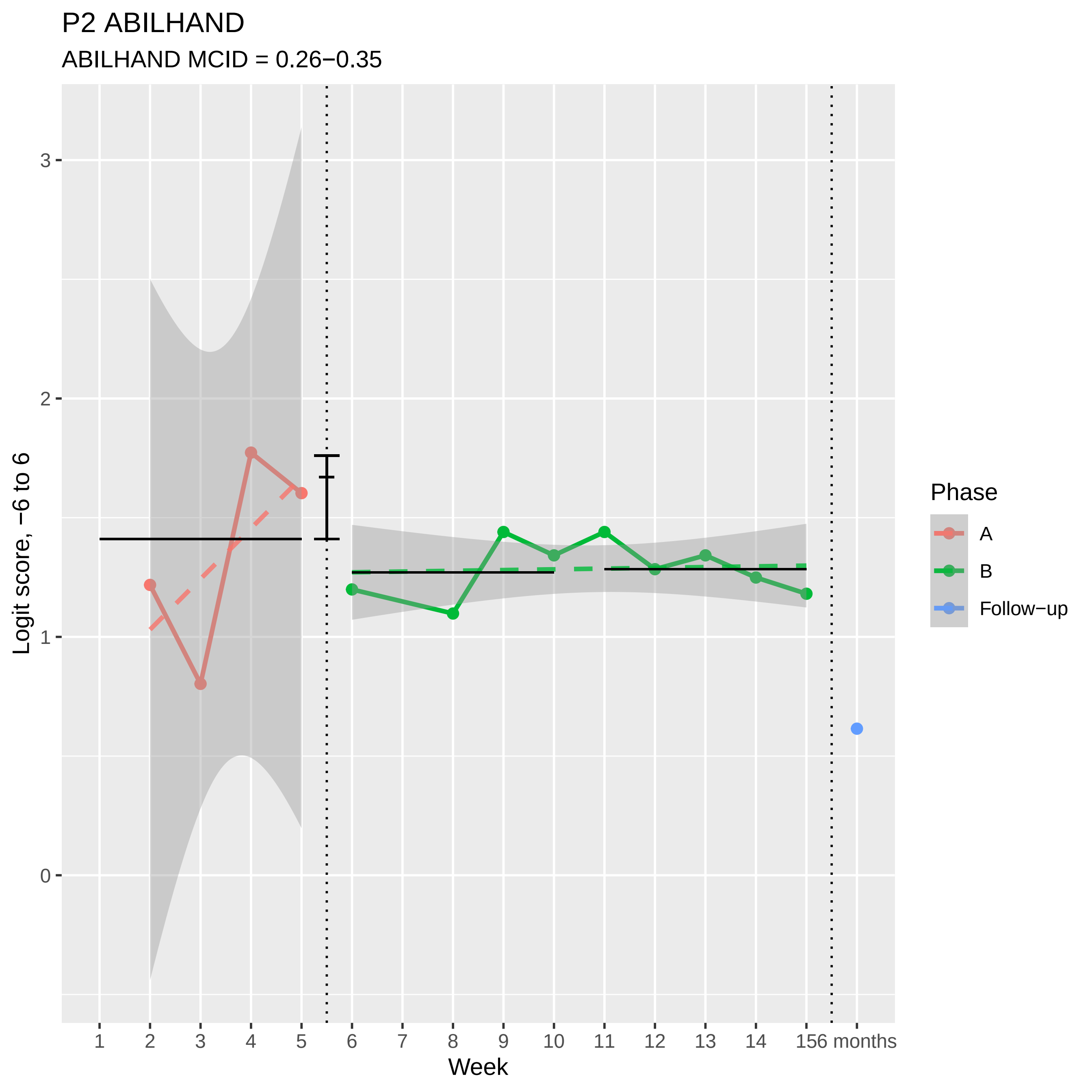

P2 ABILHAND
ABILHAND MCID = 0.26−0.35
Logit score, −6 to 6
3
2
1
0
1
2
3
4
5
6
7
8
9
10
11
12
13
14
15
6 months
Week
Phase
A
B
Follow−up

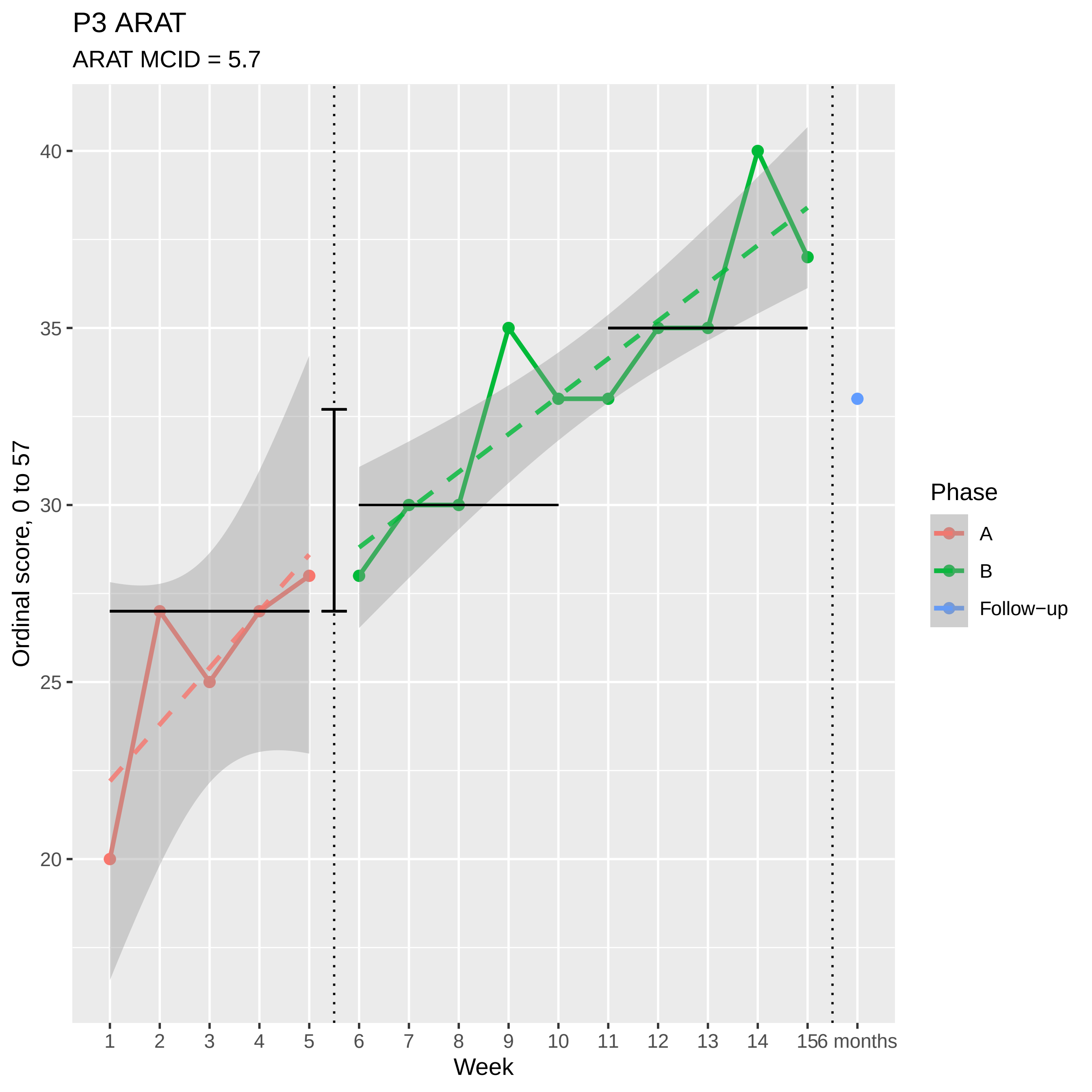

P3 ARAT
ARAT MCID = 5.7
Ordinal score, 0 to 57
40
35
30
25
20
1
2
3
4
5
6
7
8
9
10
11
12
13
14
15
6 months
Week
Phase
A
B
Follow−up

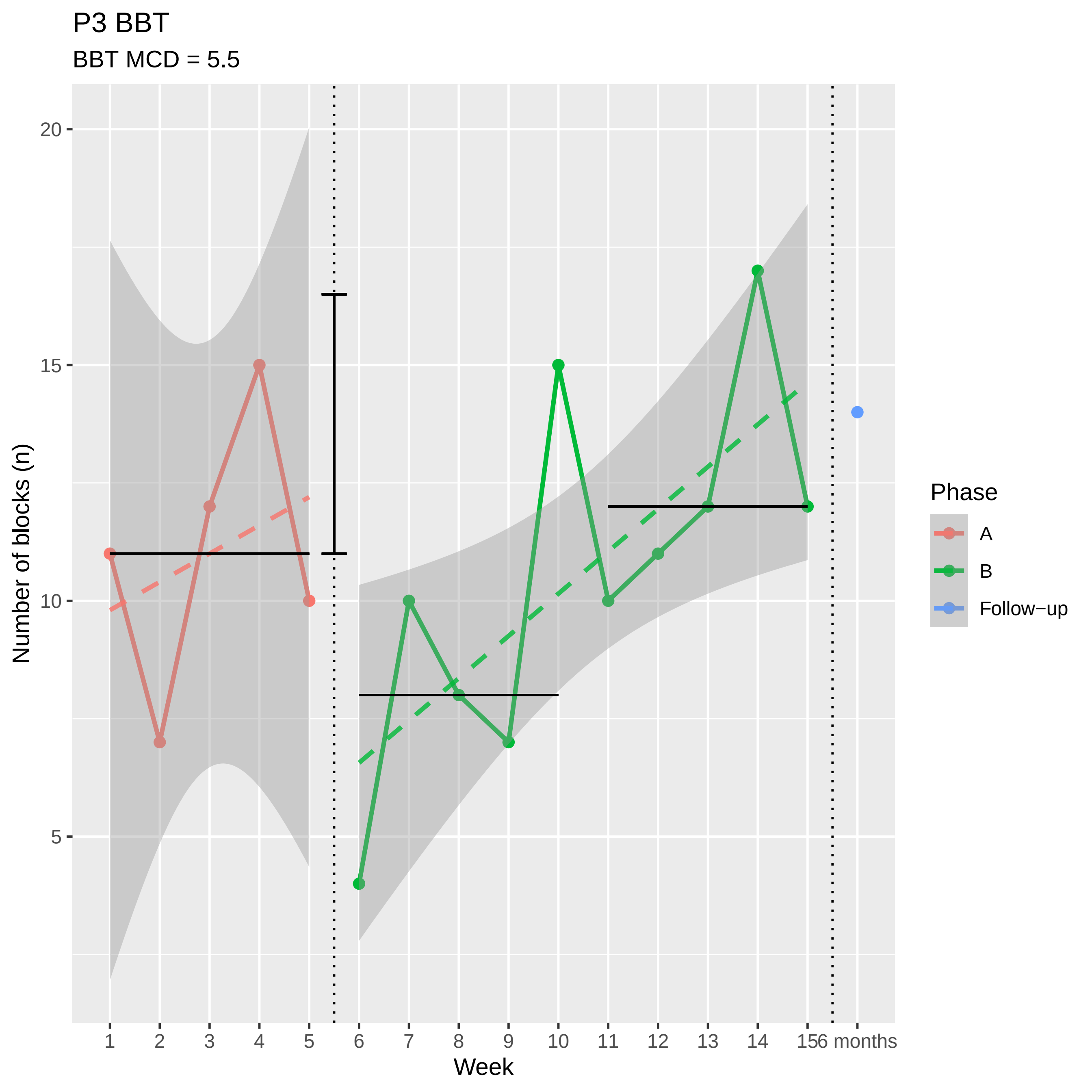

P3 BBT
BBT MCD = 5.5
Number of blocks (n)
20
15
10
5
1
2
3
4
5
6
7
8
9
10
11
12
13
14
15
6 months
Week
Phase
A
B
Follow−up

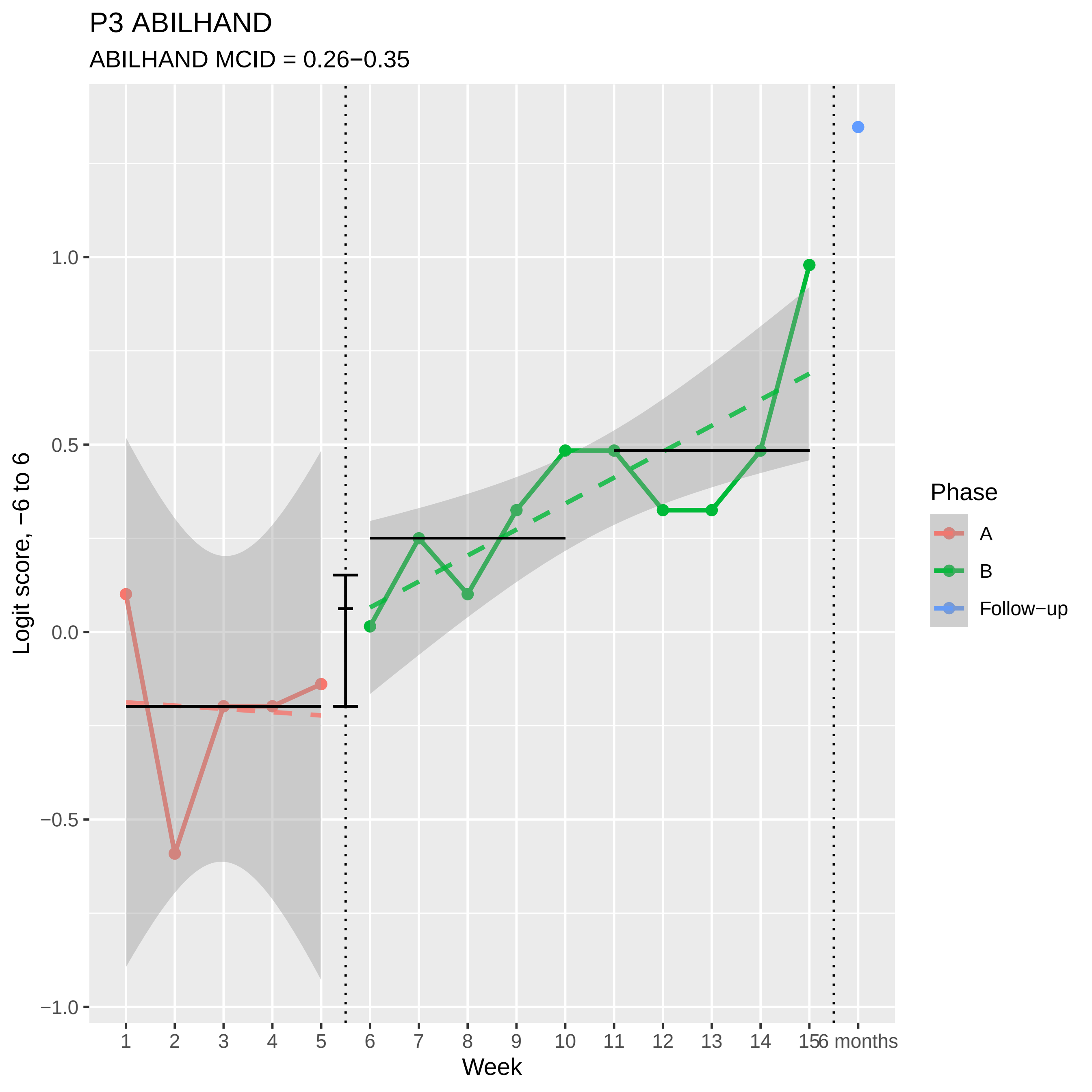

P3 ABILHAND
ABILHAND MCID = 0.26−0.35
Logit score, −6 to 6
1.0
0.5
0.0
−0.5
−1.0
1
2
3
4
5
6
7
8
9
10
11
12
13
14
15
6 months
Week
Phase
A
B
Follow−up

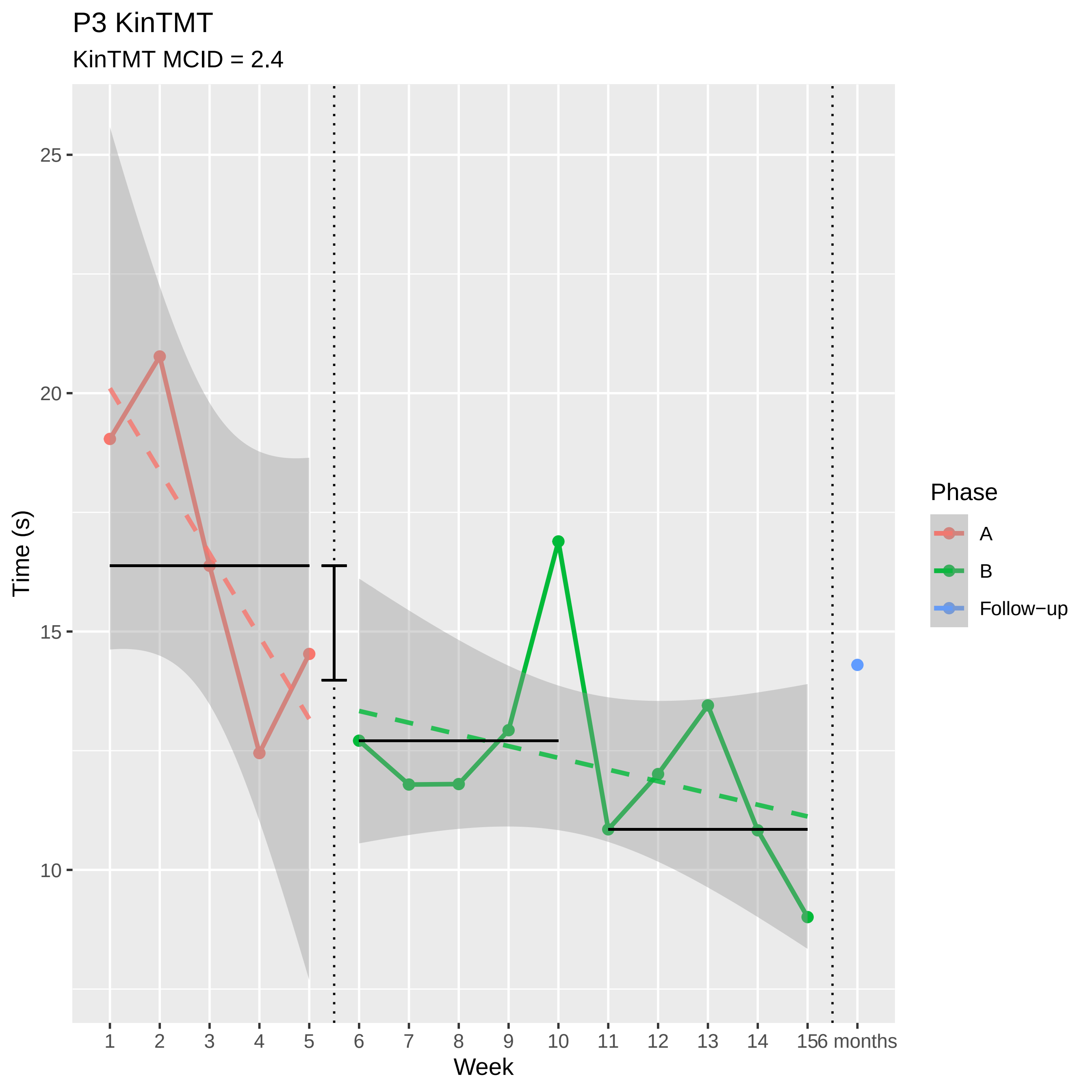
P3 KinTMT
KinTMT MCID = 2.4
Time (s)
25
20
15
10
1
2
3
4
5
6
7
8
9
10
11
12
13
14
15
6 months
Week
Phase
A
B
Follow−up

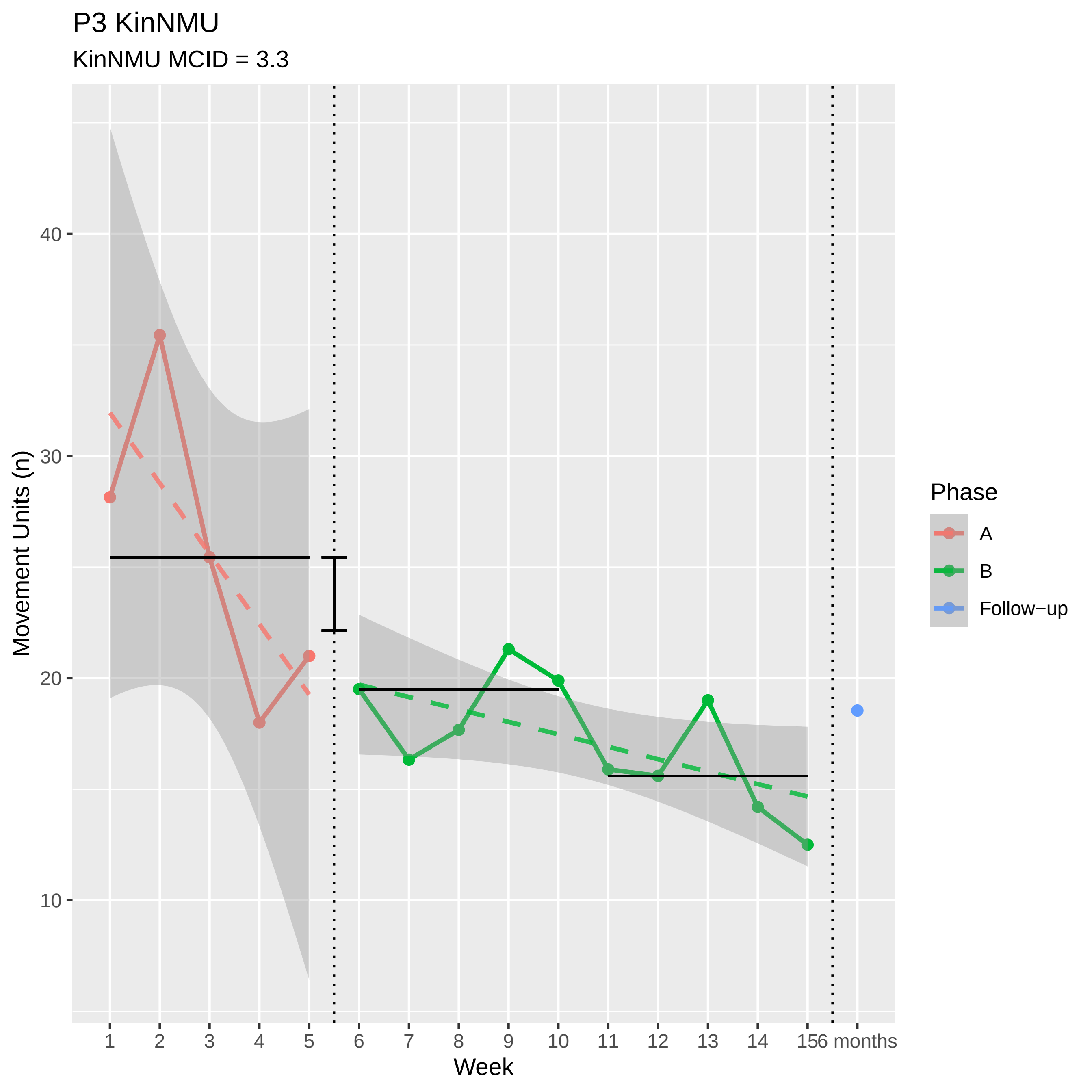

P3 KinNMU
KinNMU MCID = 3.3
Movement Units (n)
10
20
30
40
Week
1
2
3
4
5
6
7
8
9
10
11
12
13
14
15
6 months
Phase
A
B
Follow−up

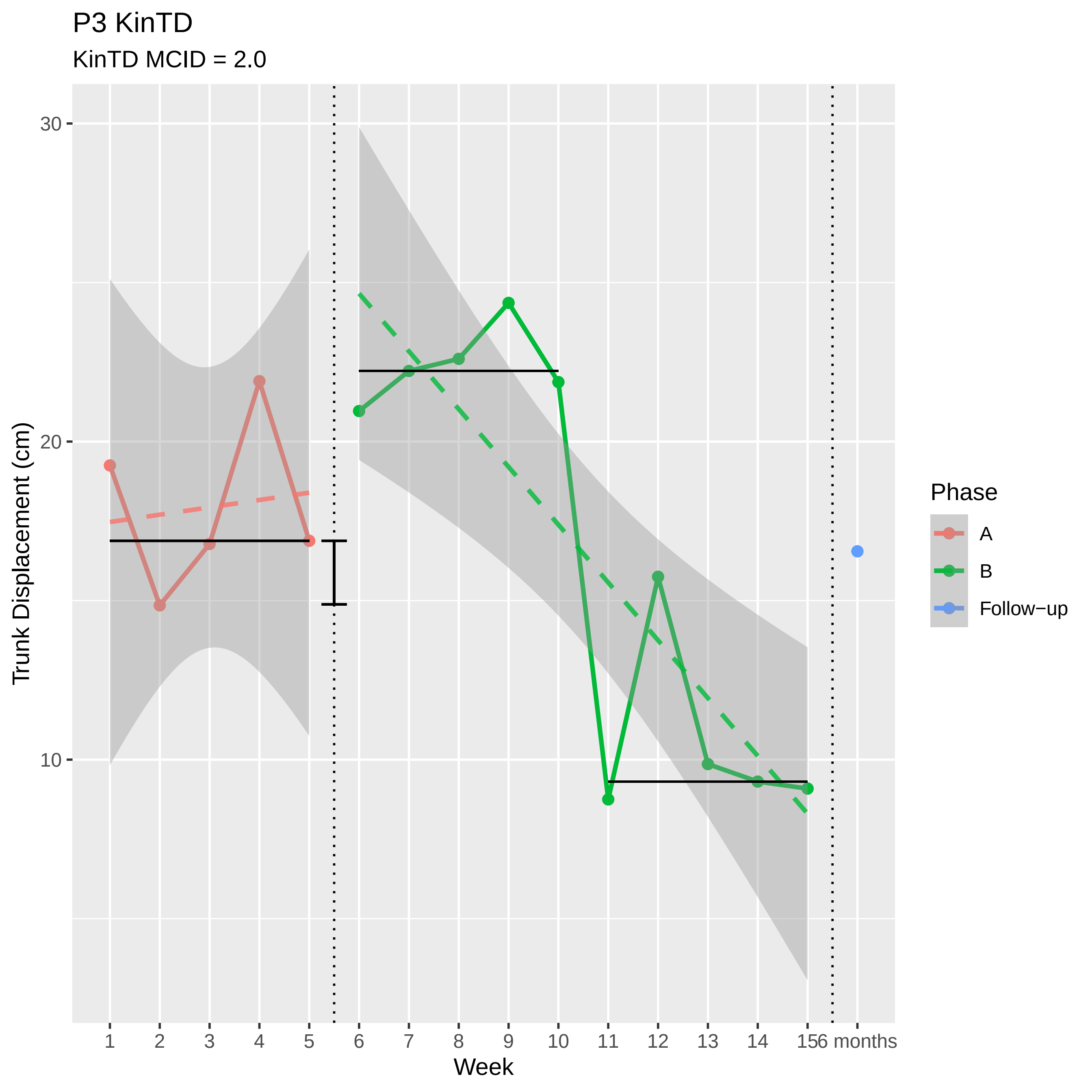

P3 KinTD
KinTD MCID = 2.0
Trunk Displacement (cm)
30
20
10
1
2
3
4
5
6
7
8
9
10
11
12
13
14
15
6 months
Week
Phase
A
B
Follow−up

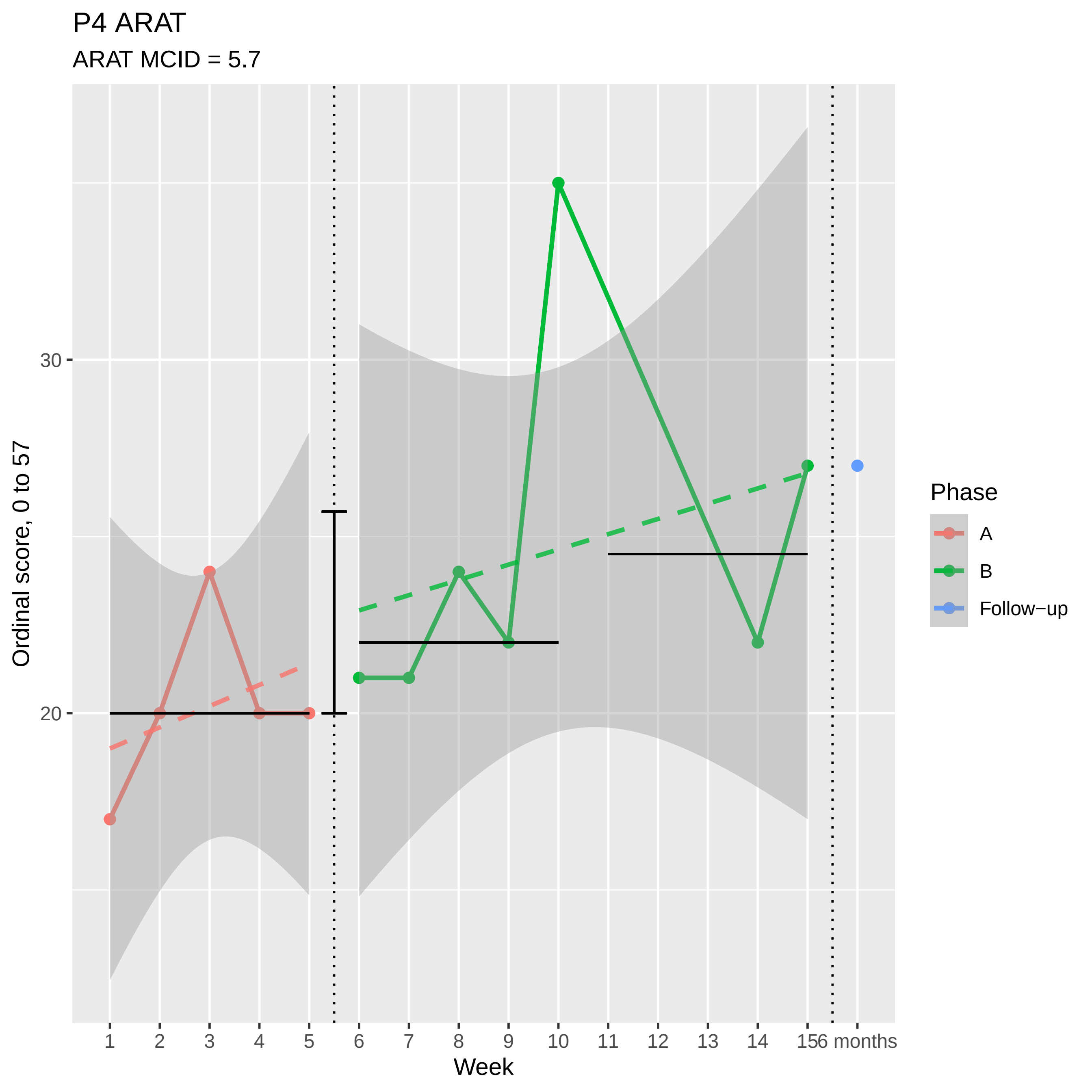
P4 ARAT
ARAT MCID = 5.7
Ordinal score, 0 to 57
30
20
Week
1
2
3
4
5
6
7
8
9
10
11
12
13
14
15
6 months
Phase
A
B
Follow−up

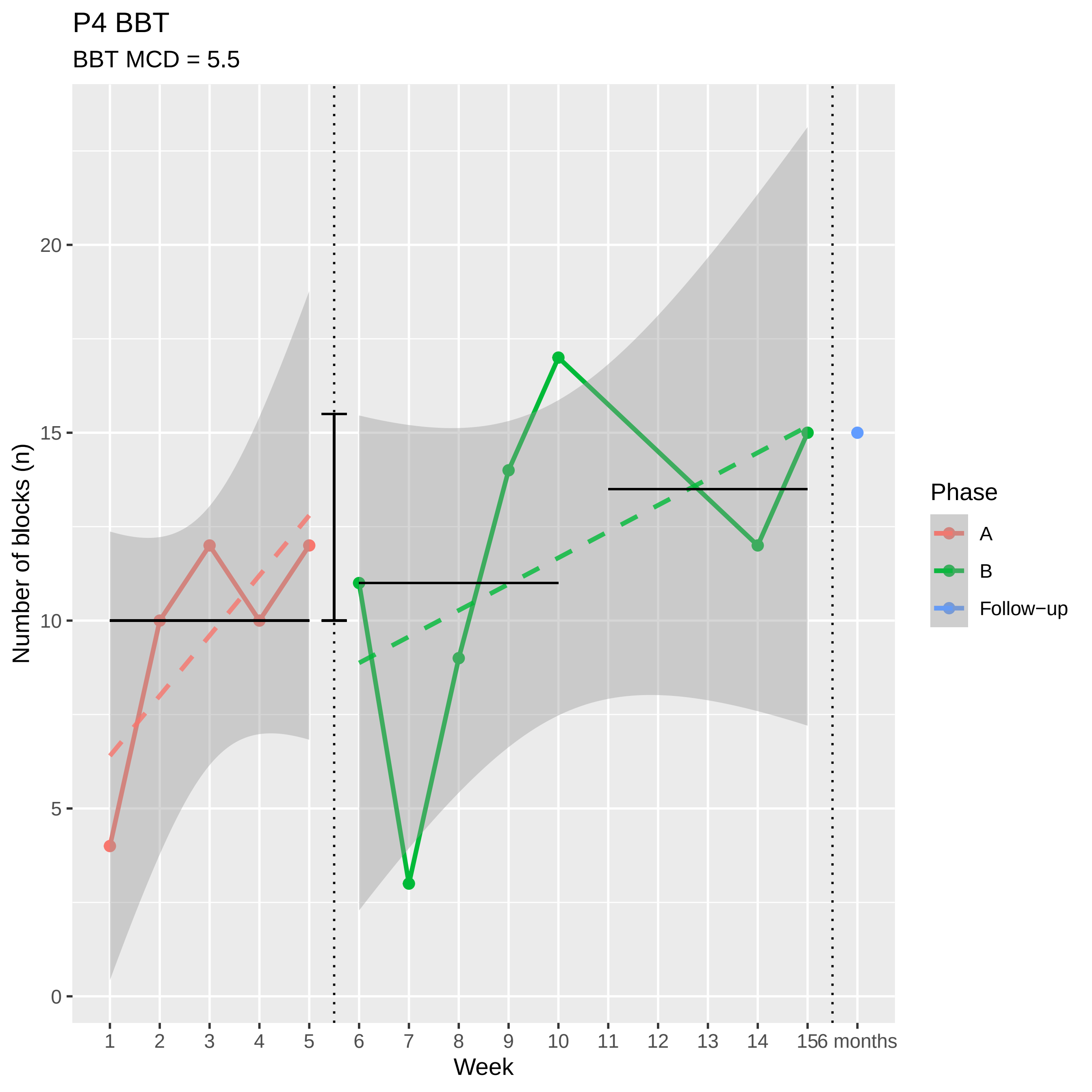
P4 BBT
BBT MCD = 5.5
Number of blocks (n)
0
5
10
15
20
1
2
3
4
5
6
7
8
9
10
11
12
13
14
15
6 months
Week
Phase
A
B
Follow−up

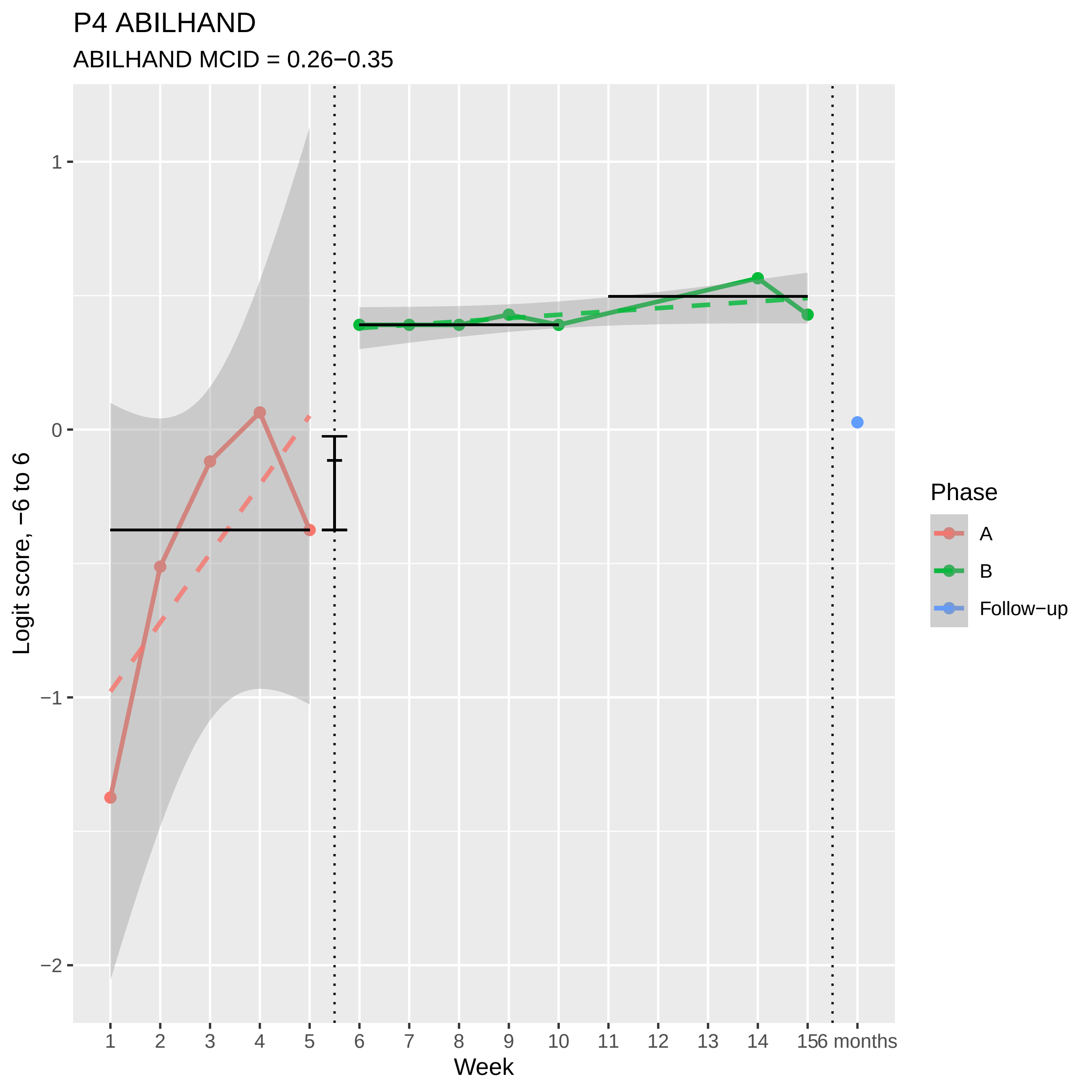

P4 ABILHAND
ABILHAND MCID = 0.26−0.35
Logit score, −6 to 6
1
0
−1
−2
1
2
3
4
5
6
7
8
9
10
11
12
13
14
15
6 months
Week
Phase
A
B
Follow−up

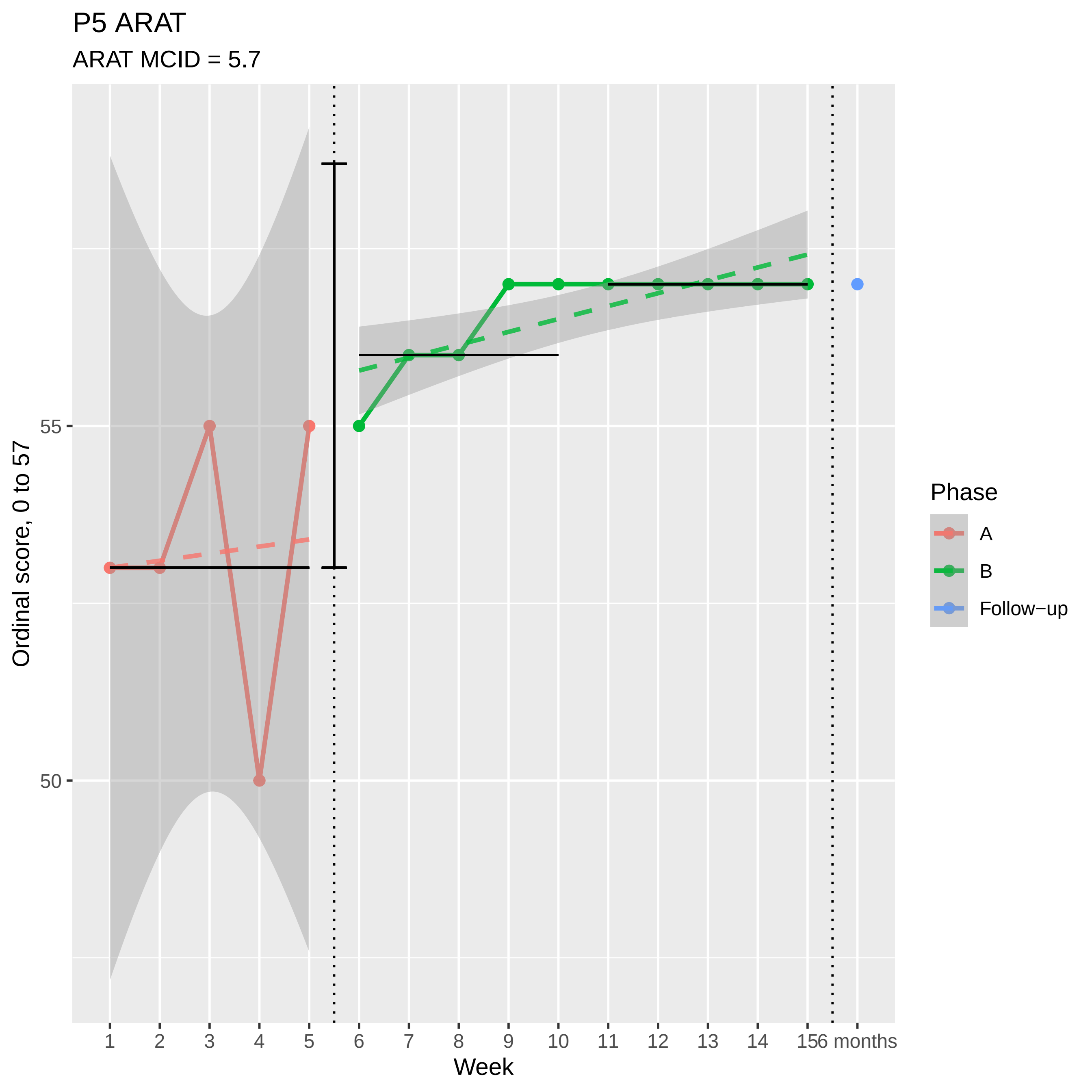

P5 ARAT
ARAT MCID = 5.7
Ordinal score, 0 to 57
55
50
1
2
3
4
5
6
7
8
9
10
11
12
13
14
15
6 months
Week
Phase
A
B
Follow−up

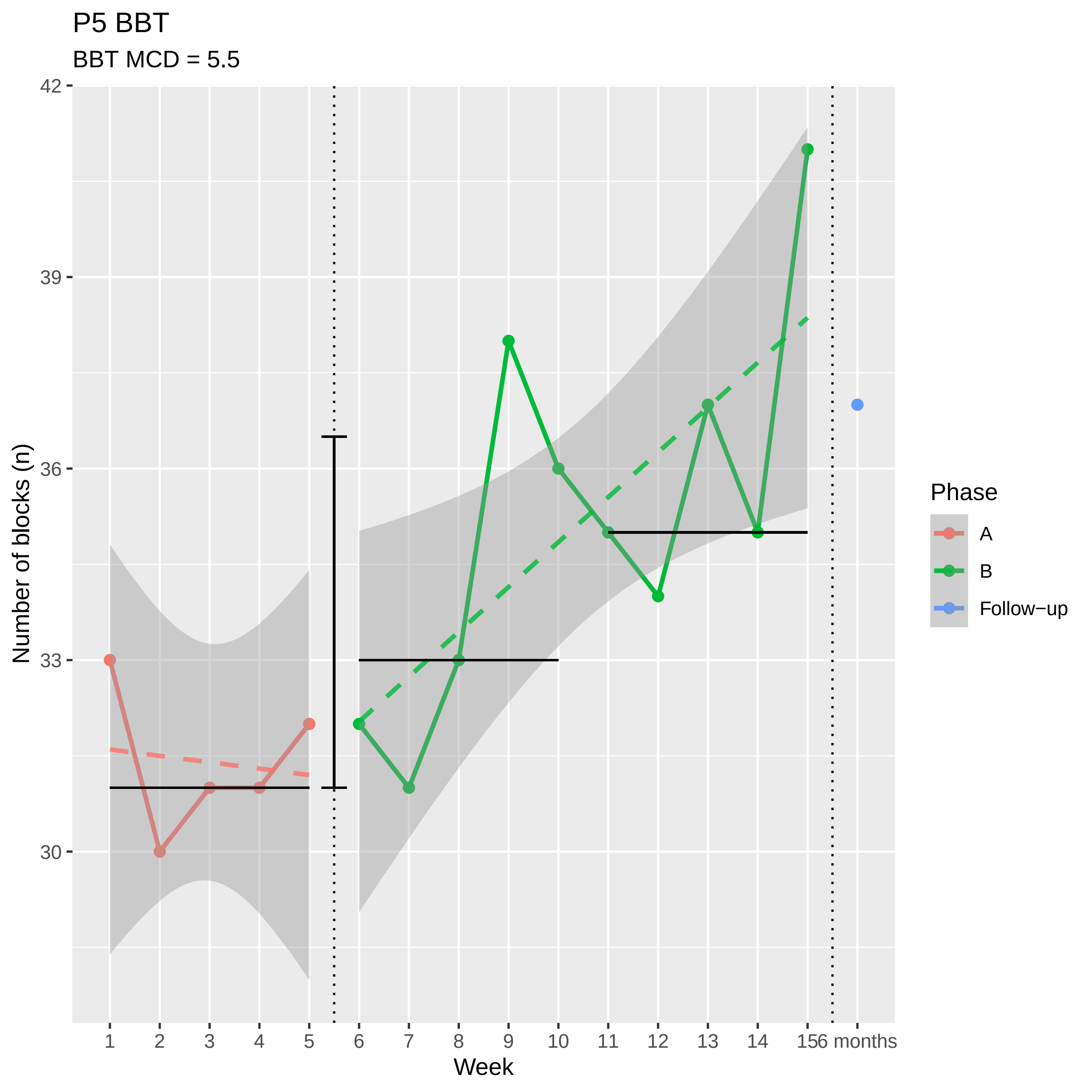

P5 BBT
BBT MCD = 5.5
42
39
36
33
30
Number of blocks (n)
1
2
3
4
5
6
7
8
9
10
11
12
13
14
15
6 months
Week
Phase
A
B
Follow−up

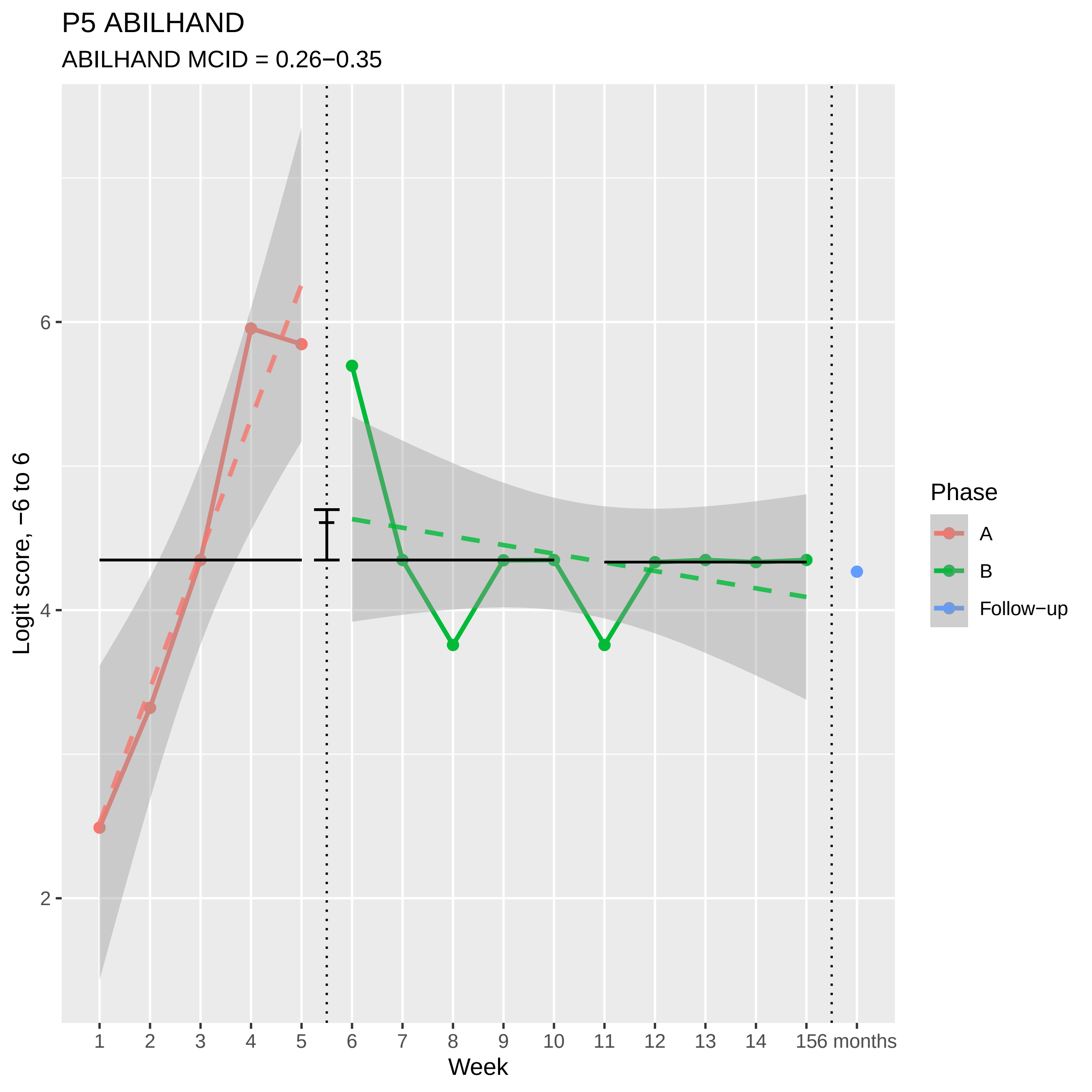
P5 ABILHAND
ABILHAND MCID = 0.26−0.35
Logit score, −6 to 6
6
4
2
1
2
3
4
5
6
7
8
9
10
11
12
13
14
15
6 months
Week
Phase
A
B
Follow−up

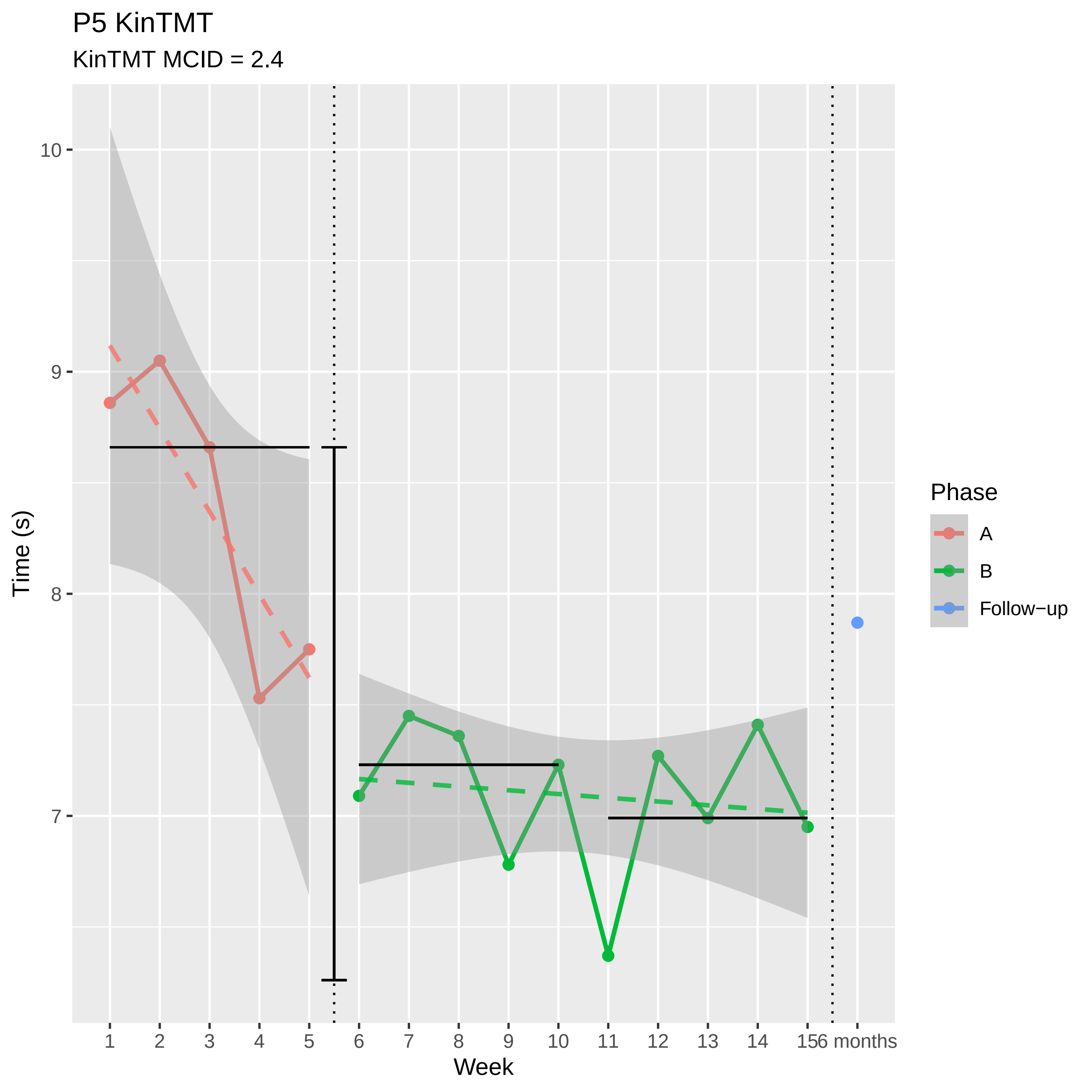
P5 KinTMT
KinTMT MCID = 2.4
Time (s)
10
9
8
7
1
2
3
4
5
6
7
8
9
10
11
12
13
14
15
6 months
Week
Phase
A
B
Follow−up

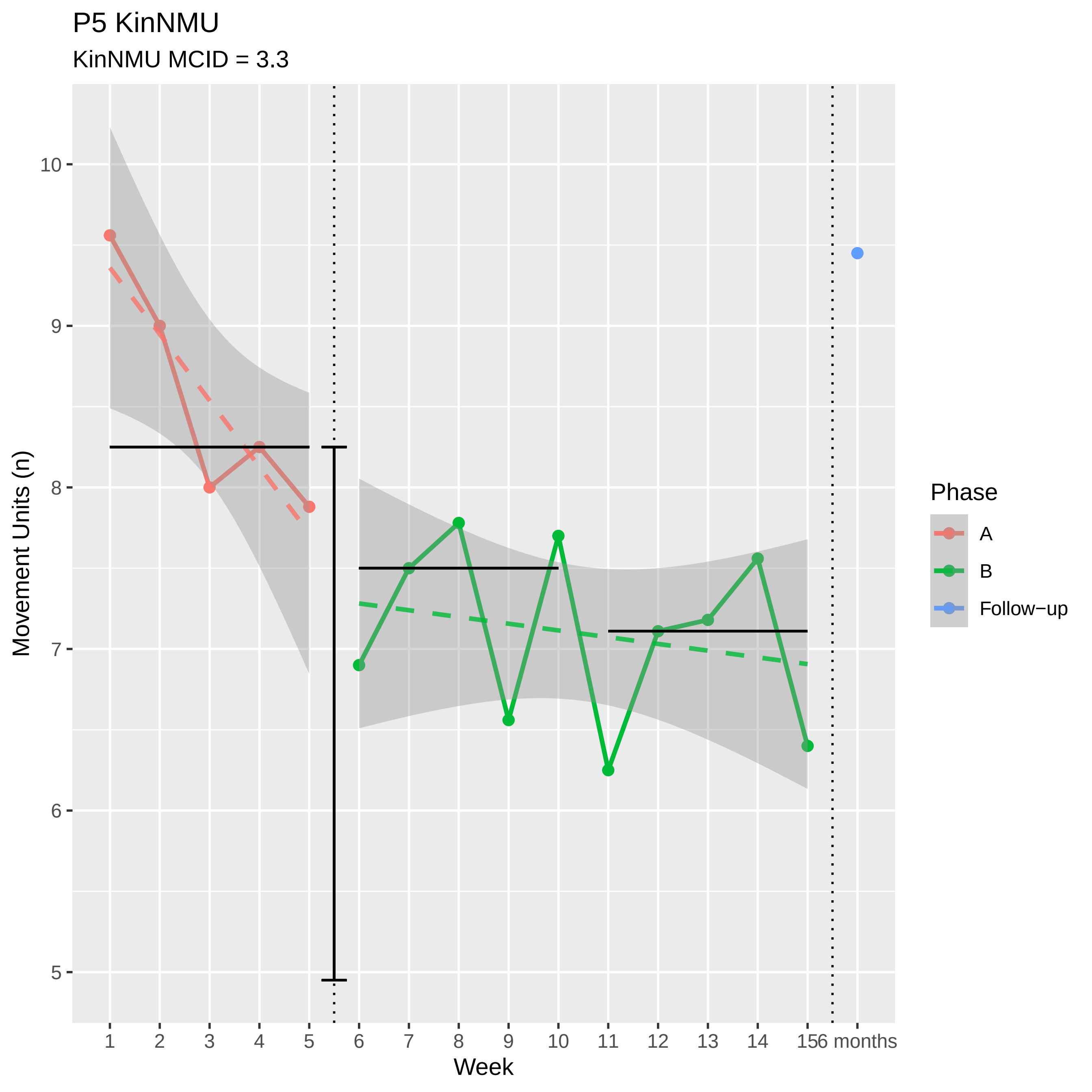
P5 KinNMU
KinNMU MCID = 3.3
Movement Units (n)
10
9
8
7
6
5
1
2
3
4
5
6
7
8
9
10
11
12
13
14
15
6 months
Week
Phase
A
B
Follow−up

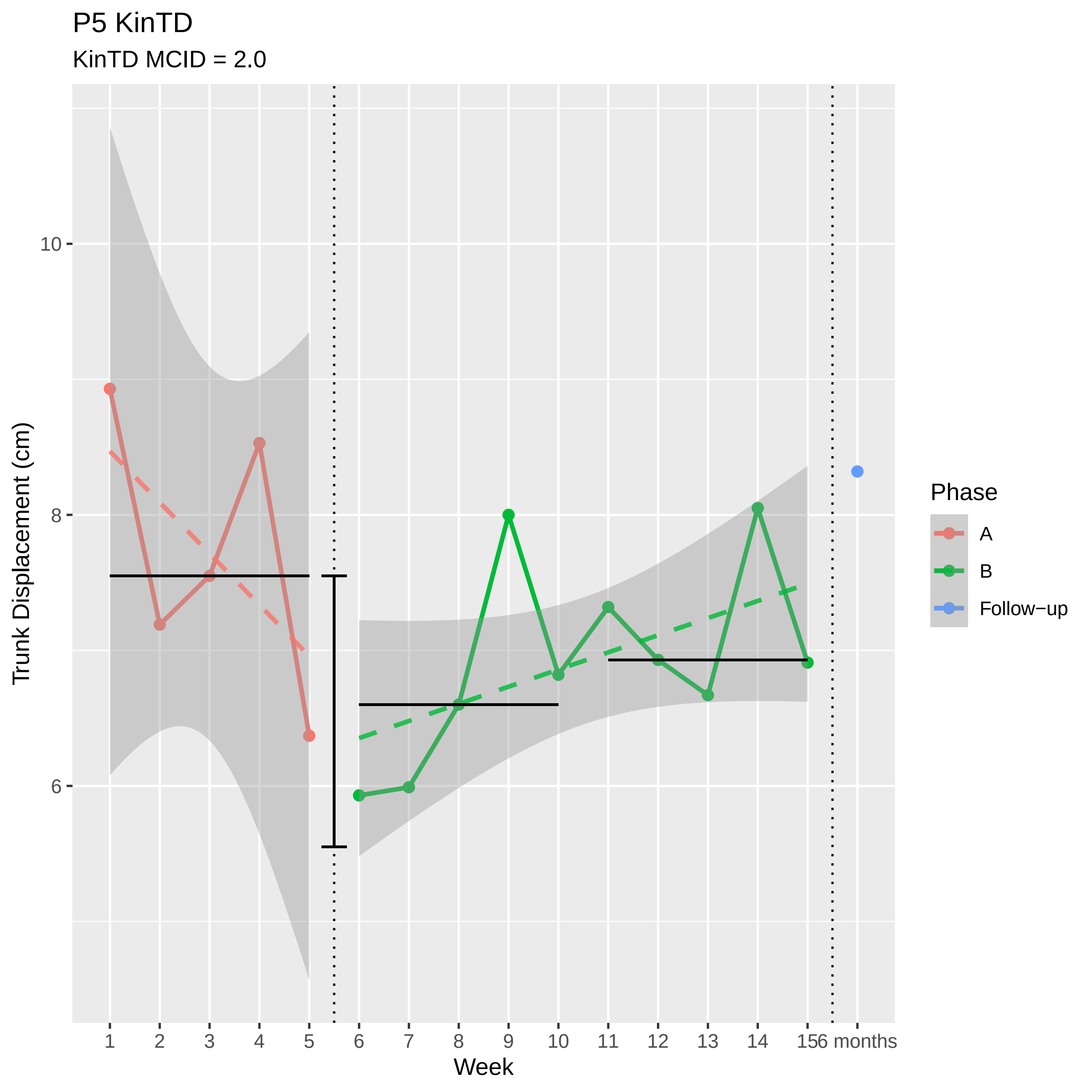
P5 KinTD
KinTD MCID = 2.0
Trunk Displacement (cm)
10
8
6
1
2
3
4
5
6
7
8
9
10
11
12
13
14
15
6 months
Week
Phase
A
B
Follow−up

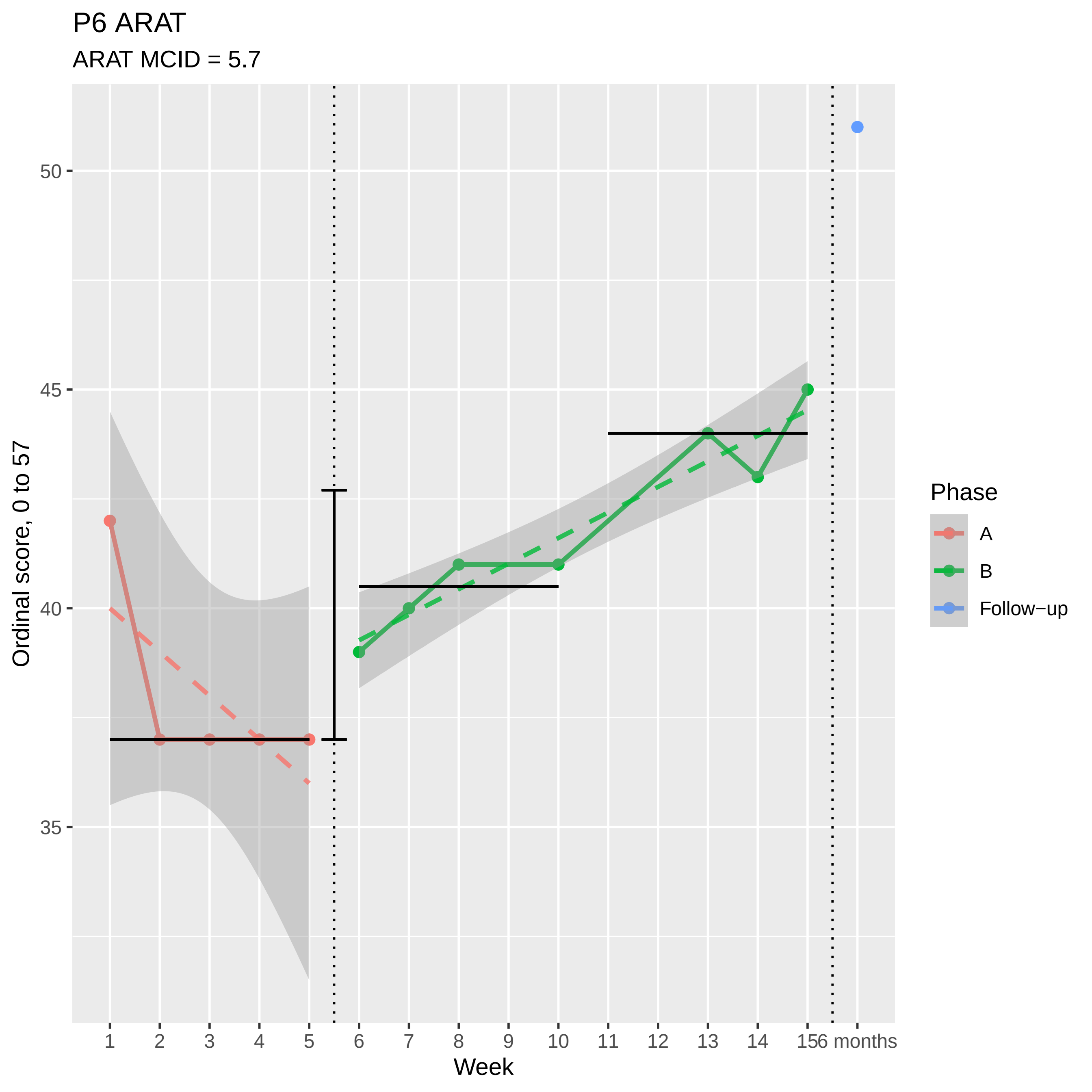
P6 ARAT
ARAT MCID = 5.7
Ordinal score, 0 to 57
50
45
40
35
1
2
3
4
5
6
7
8
9
10
11
12
13
14
15
6 months
Week
Phase
A
B
Follow−up

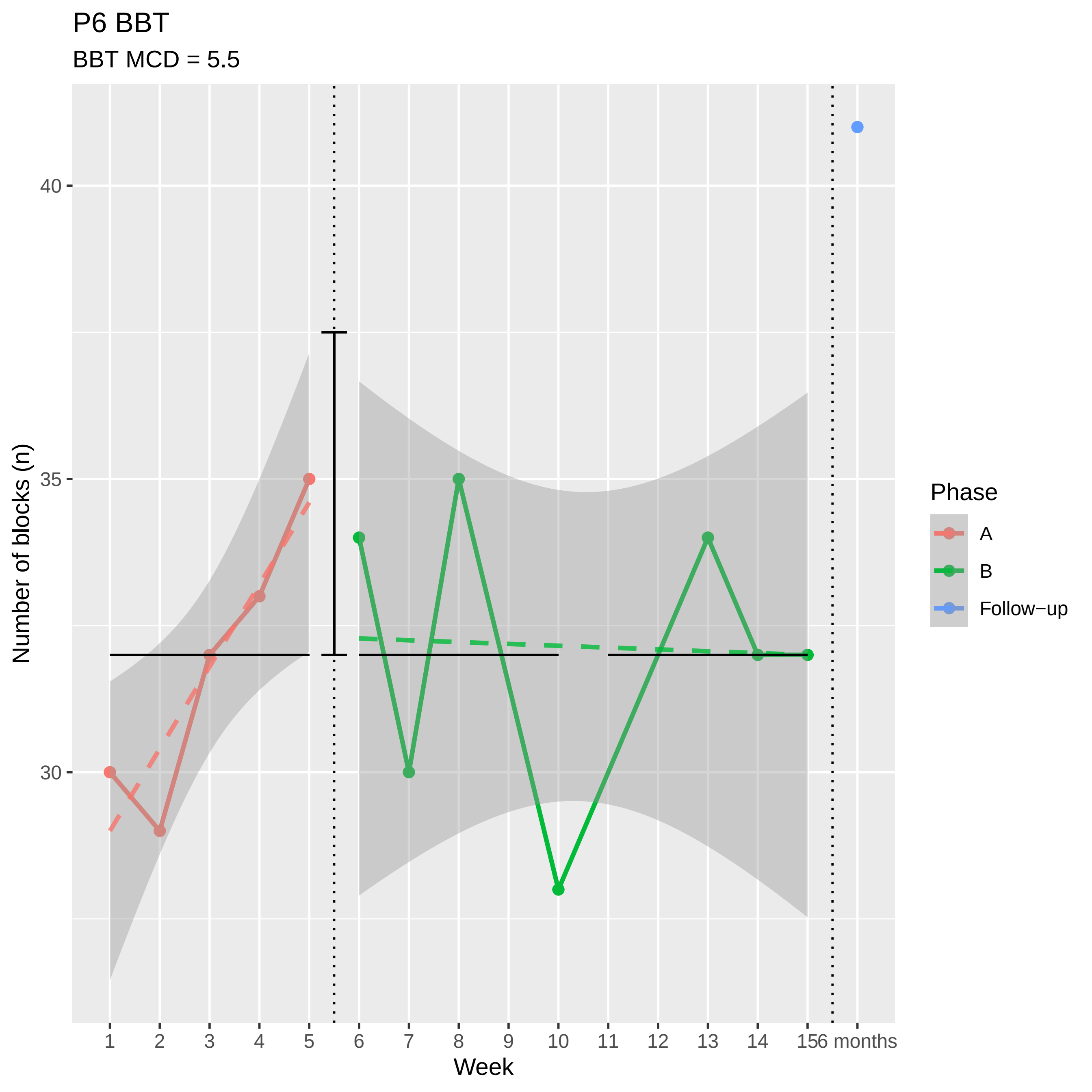
P6 BBT
BBT MCD = 5.5
Number of blocks (n)
40
35
30
Week
1
2
3
4
5
6
7
8
9
10
11
12
13
14
15
6 months
Phase
A
B
Follow−up

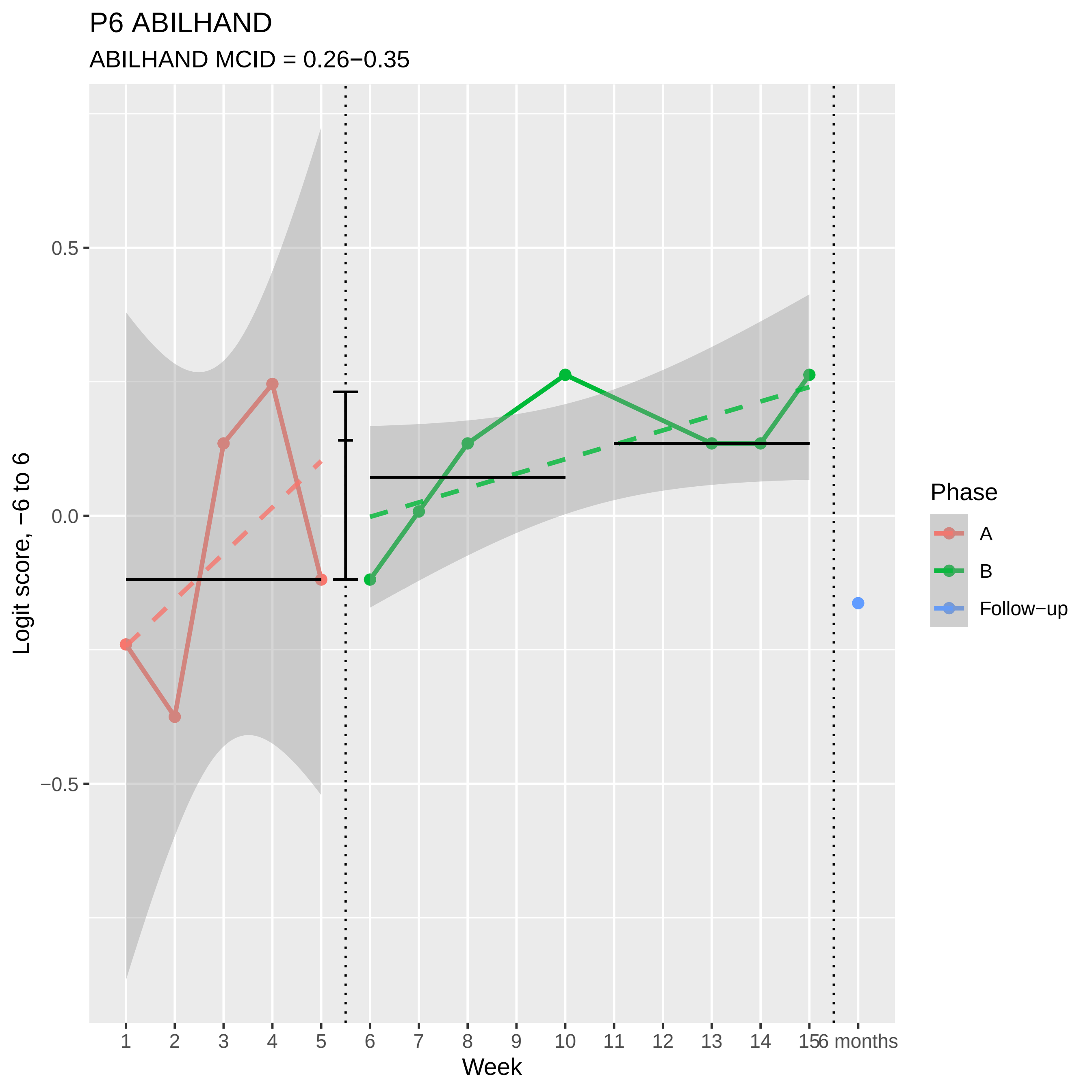

P6 ABILHAND
ABILHAND MCID = 0.26−0.35
Logit score, −6 to 6
0.5
0.0
−0.5
Week
1
2
3
4
5
6
7
8
9
10
11
12
13
14
15
6 months
Phase
A
B
Follow−up

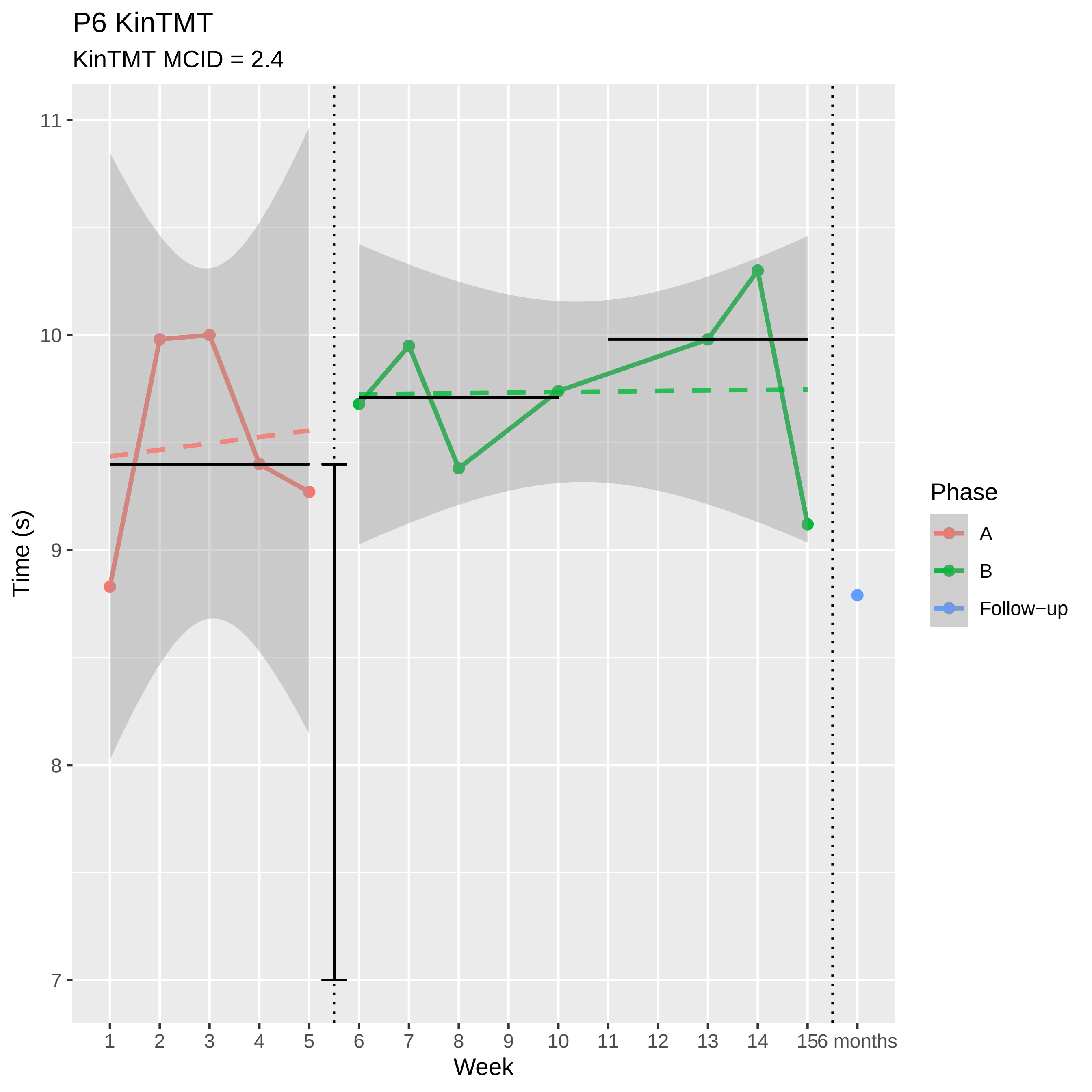

P6 KinTMT
KinTMT MCID = 2.4
Time (s)
11
10
9
8
7
Week
1
2
3
4
5
6
7
8
9
10
11
12
13
14
15
6 months
Phase
A
B
Follow−up

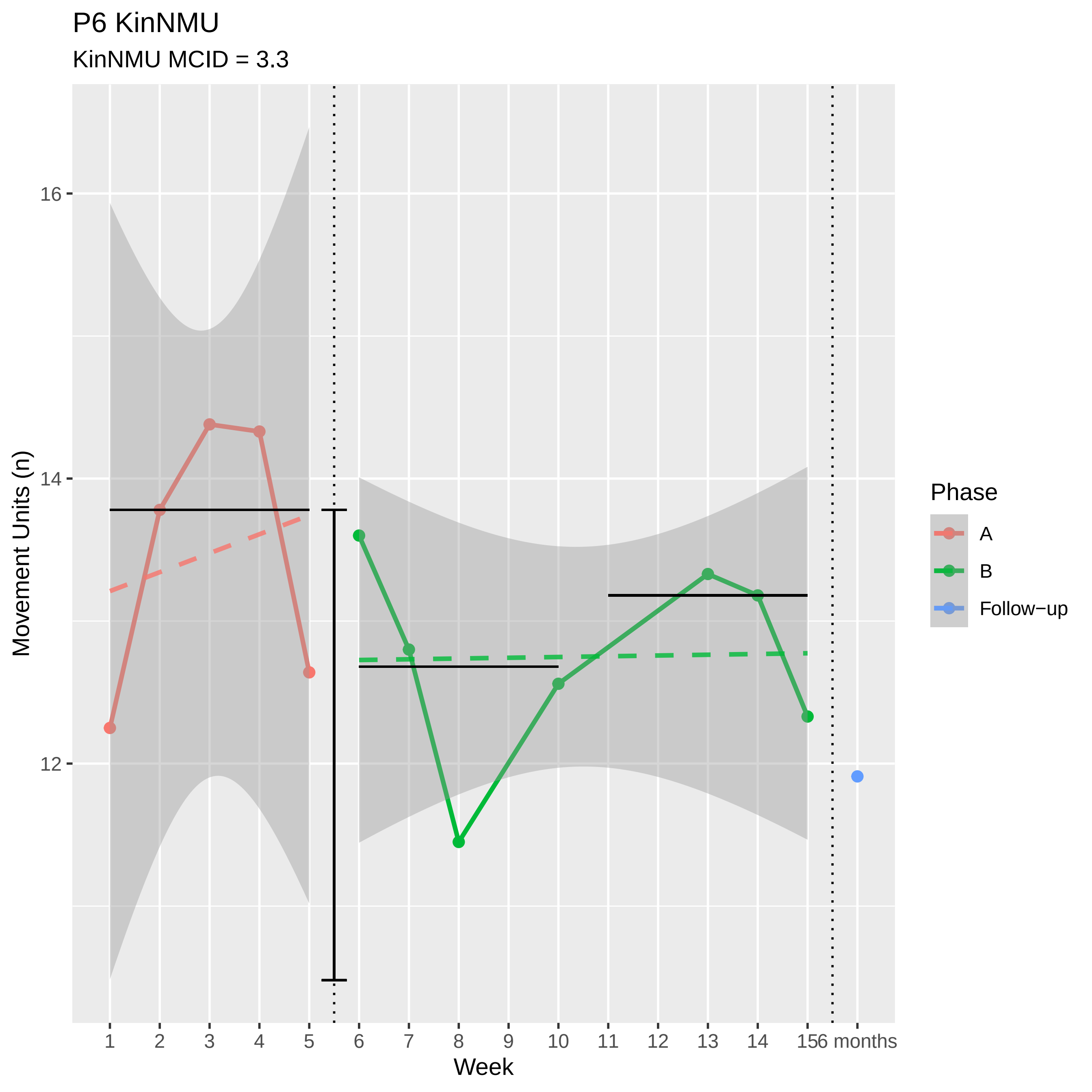
P6 KinNMU
KinNMU MCID = 3.3
Movement Units (n)
16
14
12
Week
1
2
3
4
5
6
7
8
9
10
11
12
13
14
15
6 months
Phase
A
B
Follow−up

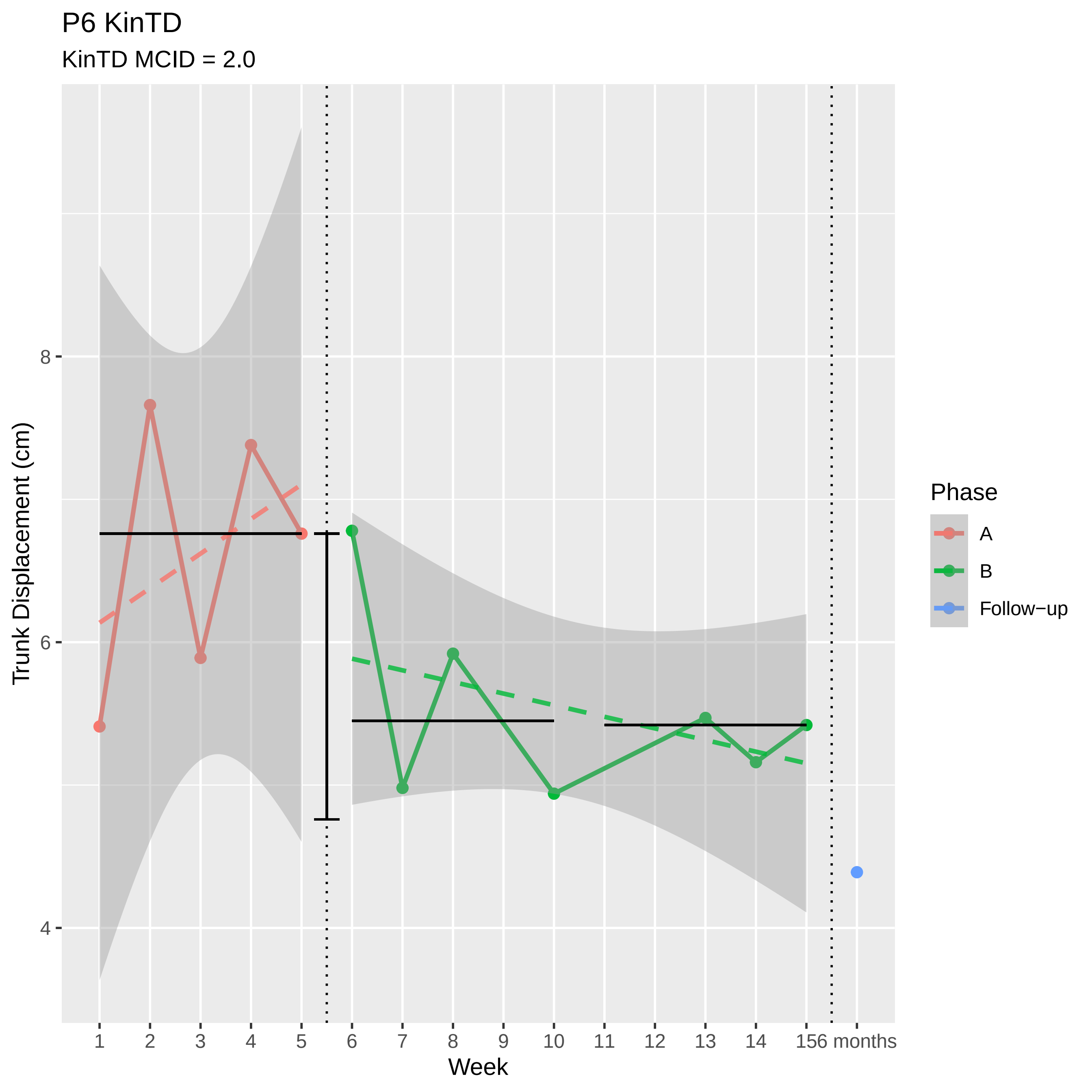
P6 KinTD
KinTD MCID = 2.0
Trunk Displacement (cm)
8
6
4
Week
1
2
3
4
5
6
7
8
9
10
11
12
13
14
15
6 months
Phase
A
B
Follow−up

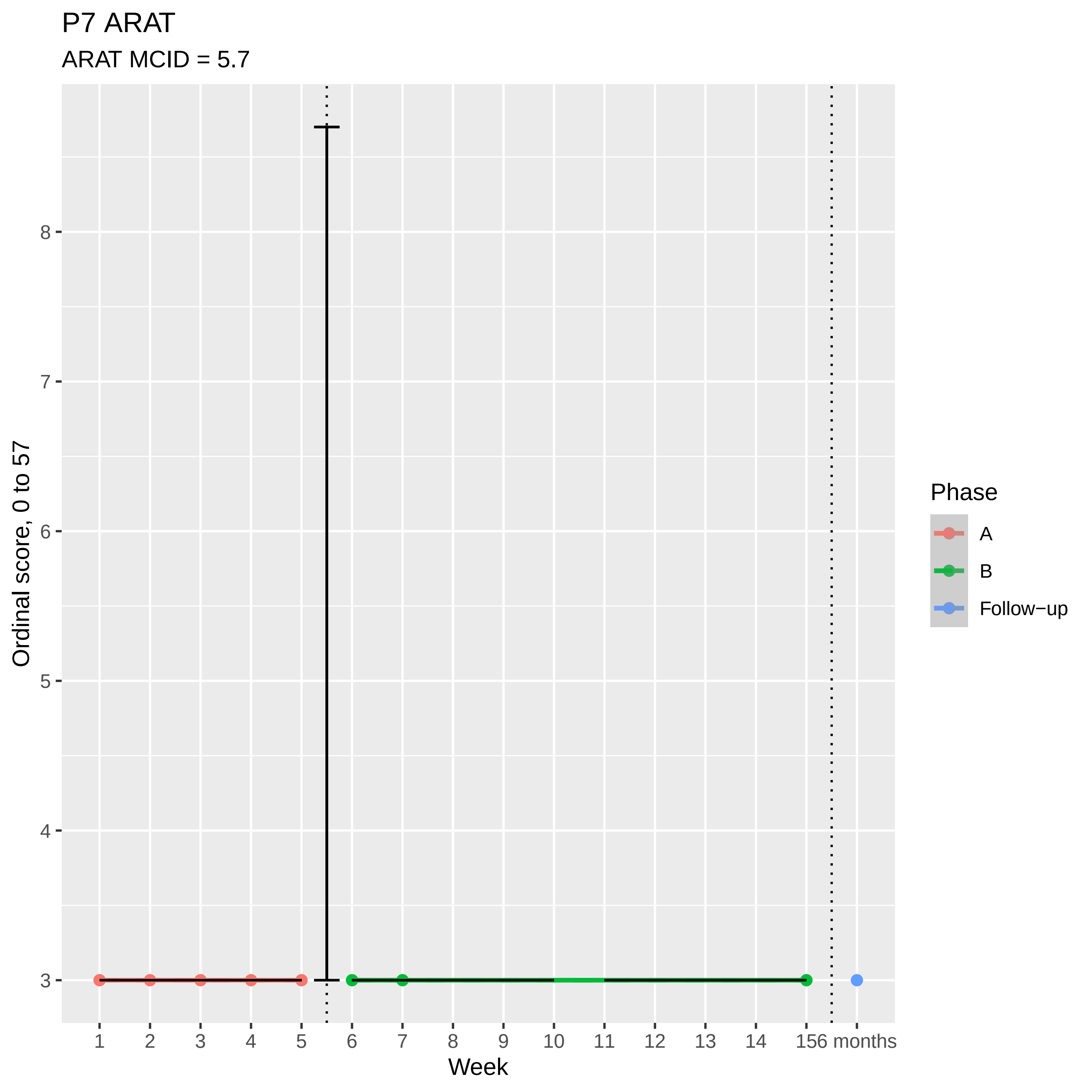
P7 ARAT
ARAT MCID = 5.7
Ordinal score, 0 to 57
3
4
5
6
7
8
Week
1
2
3
4
5
6
7
8
9
10
11
12
13
14
15
6 months
Phase
A
B
Follow−up

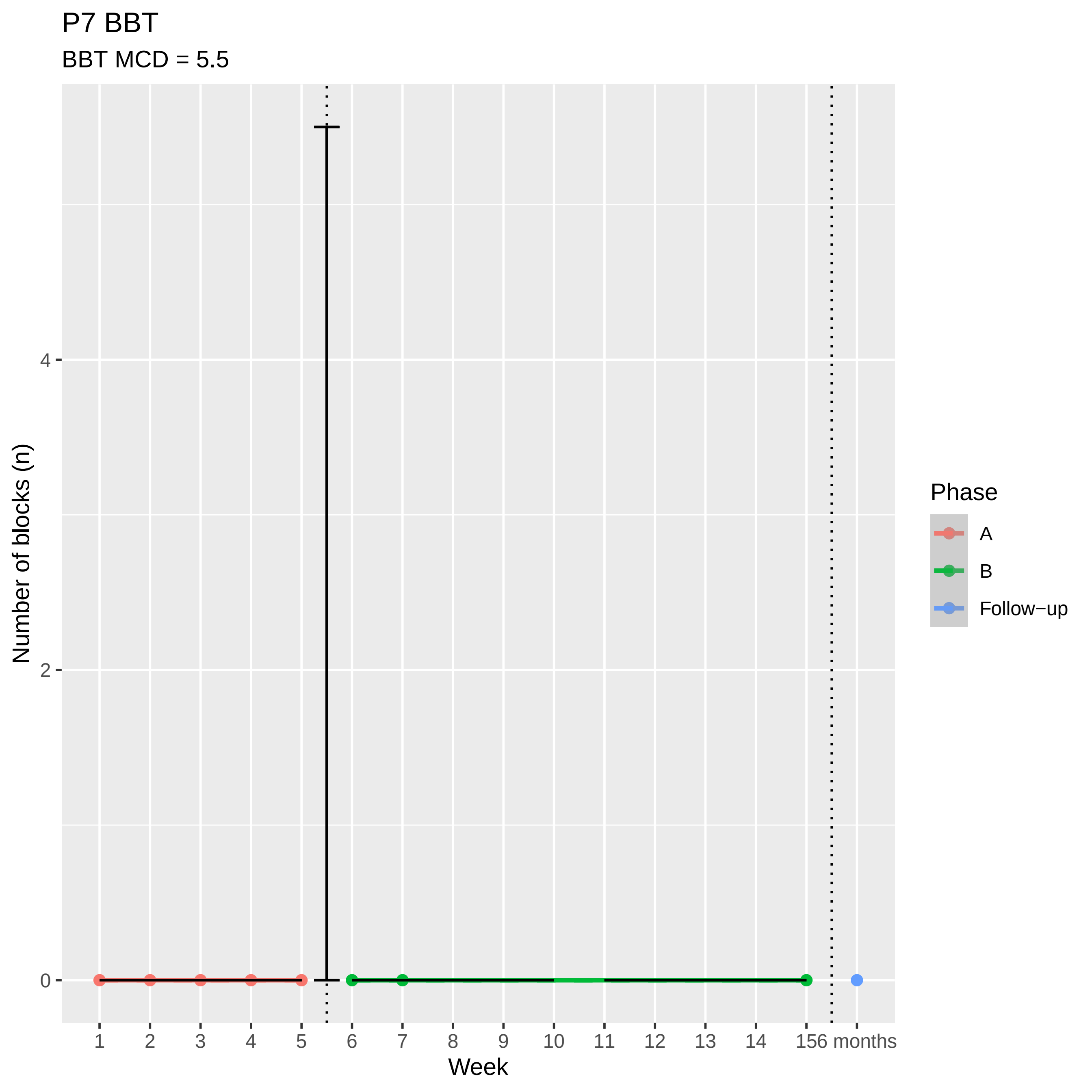
P7 BBT
BBT MCD = 5.5
Number of blocks (n)
0
2
4
1
2
3
4
5
6
7
8
9
10
11
12
13
14
15
6 months
Week
Phase
A
B
Follow−up

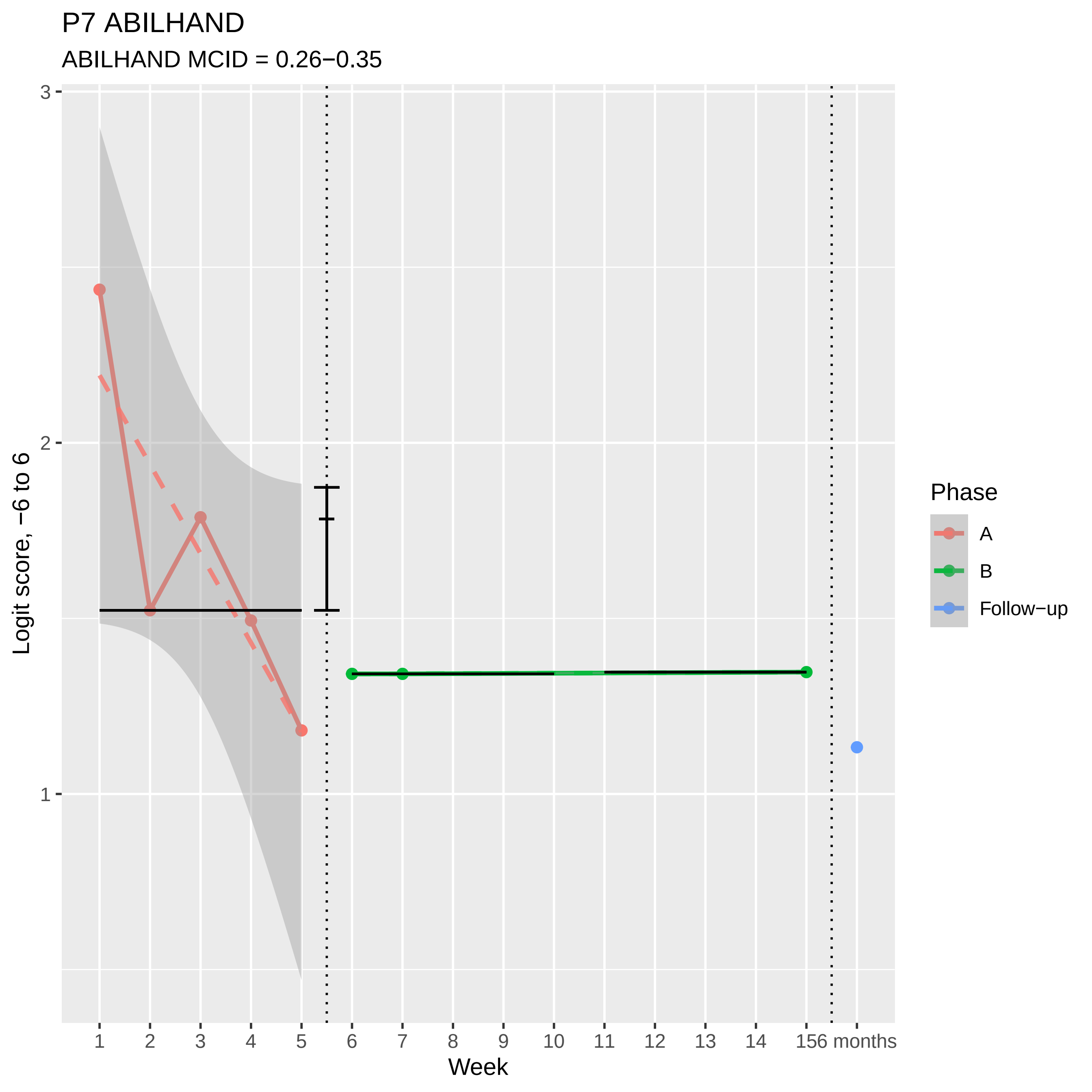

P7 ABILHAND
ABILHAND MCID = 0.26−0.35
Logit score, −6 to 6
3
2
1
Week
1
2
3
4
5
6
7
8
9
10
11
12
13
14
15
6 months
Phase
A
B
Follow−up
